# Application of an angiogenesis-related genes risk model in lung adenocarcinoma prognosis and immunotherapy

**DOI:** 10.3389/fgene.2023.1092968

**Published:** 2023-02-01

**Authors:** Jinsong Wang, Xue Cui, Yiming Weng, Jiayan Wei, Xinyi Chen, Peiwei Wang, Tong Wang, Jian Qin, Min Peng

**Affiliations:** ^1^ Department of Oncology, Renmin Hospital of Wuhan University, Wuhan, China; ^2^ Central Laboratory, Renmin Hospital, Wuhan University, Wuhan, China

**Keywords:** angiogenesis-related genes, risk model, lung adenocarcinoma, prognosis, immunotherapy

## Abstract

Lung adenocarcinoma (LUAD) is an essential pathological subtype of non-small cell lung cancer and offers a severe problem for worldwide public health. There is mounting proof that angiogenesis is a crucial player in LUAD progression. Consequently, the purpose of this research was to construct a novel LUAD risk assessment model based on genetic markers related to angiogenesis. We accessed The Cancer Genome Atlas (TCGA) and Gene Expression Omnibus (GEO) databases for LUAD mRNA sequencing data and clinical information. Based on machine algorithms and bioinformatics, angiogenic gene-related risk scores (RS) were calculated. Patients in the high-risk category had a worse prognosis (*p* < 0.001) in the discovery TCGA cohort, and the results were confirmed by these three cohorts (validation TCGA cohort, total TCGA cohort, and GSE68465 cohort). Moreover, risk scores for genes involved in angiogenesis were independent risk factors for lung cancer in all four cohorts. The low-risk group was associated with better immune status and lower tumor mutational load. In addition, the somatic mutation study revealed that the low-risk group had a lower mutation frequency than the high-risk group. According to an analysis of tumor stem cell infiltration, HLA expression, and TIDE scores, the low-risk group had higher TIDE scores and HLA expression levels than the high-risk group, and the amount of tumor stem cell infiltration correlated with the risk score. In addition, high-risk groups may benefit from immune checkpoint inhibitors and targeted therapies. In conclusion, we developed an angiogenesis-related gene risk model to predict the prognosis of LUAD patients, which may aid in the classification of patients with LUAD and select medications for LUAD patients.

## 1 Introduction

Lung cancer is a global health issue and one of the top causes of morbidity and death among cancer patients, posing a severe danger to public health (Torre et al., 2012). There are several histological subtypes of lung cancer, with lung adenocarcinoma (LUAD) being the most common, accounting for around 40% of all lung malignancies (Ruiz-Cordero and Devine, 2020). The LUAD survival rate is just 4%–17% (Hirsch et al., 2017). In China, lung cancer prevention is complicated by high smoking rates and exposure to secondhand smoke (Qiu et al., 2021). Significant progress has been achieved in recent years as research into possible treatment targets for LUAD has continued to increase (Hirsch et al., 2017). Several immunotherapies targeting PD-1 and PD-L1 have been used clinically with significant survival benefits for LUAD patients (Steven et al., 2016). However, medication resistance and high recurrence rates continue to be the primary causes of treatment failure, resulting in unsatisfactory 5-year survival rates. To benefit more LUAD patients, there is an urgent need to identify new therapeutic targets and prognostic indicators to predict survival and guide clinical treatment in LUAD patients.

The presence of pathological angiogenesis is essential for tumor development and progression. On the one hand, these new blood vessels provide oxygen and nutrients to sustain rapid tumor growth and proliferation while helping tumor cells excrete metabolic waste. On the other hand, they provide pathways for tumor cells to enter the bloodstream and undergo distant metastasis (Viallard and Larrivée, 2017). Therefore, it is crucial for tumor cells’ survival, invasion, and metastasis (Jiang et al., 2020). Numerous activating and inhibitory factors govern tumor angiogenesis, control pathological angiogenesis, and impact patient prognosis. The factors vascular endothelial growth factor (Liu et al., 2021; Abdulazeem et al., 2022), platelet-derived growth factor (Zhang et al., 2019), and fibroblast-derived growth factor all play essential roles in angiogenesis (Eguchi and Wakabayashi, 2020). Serum VEGF promotes proliferation and migration, inhibits apoptosis, and regulates endothelial permeability (Ferrara et al., 2003). Anti-tumor angiogenic drugs such as bevacizumab (Syrigos et al., 2021) are currently used to treat lung adenocarcinoma. However, current research has focused on the impact of individual angiogenic genes on lung adenocarcinoma development and prognosis as a potential target for drug development. Few studies have integrated multiple angiogenesis-related genes by high-throughput biomarker sequencing and synthesized the relationship between these genes and lung adenocarcinoma prognosis and survival.

**FIGURE 1:**
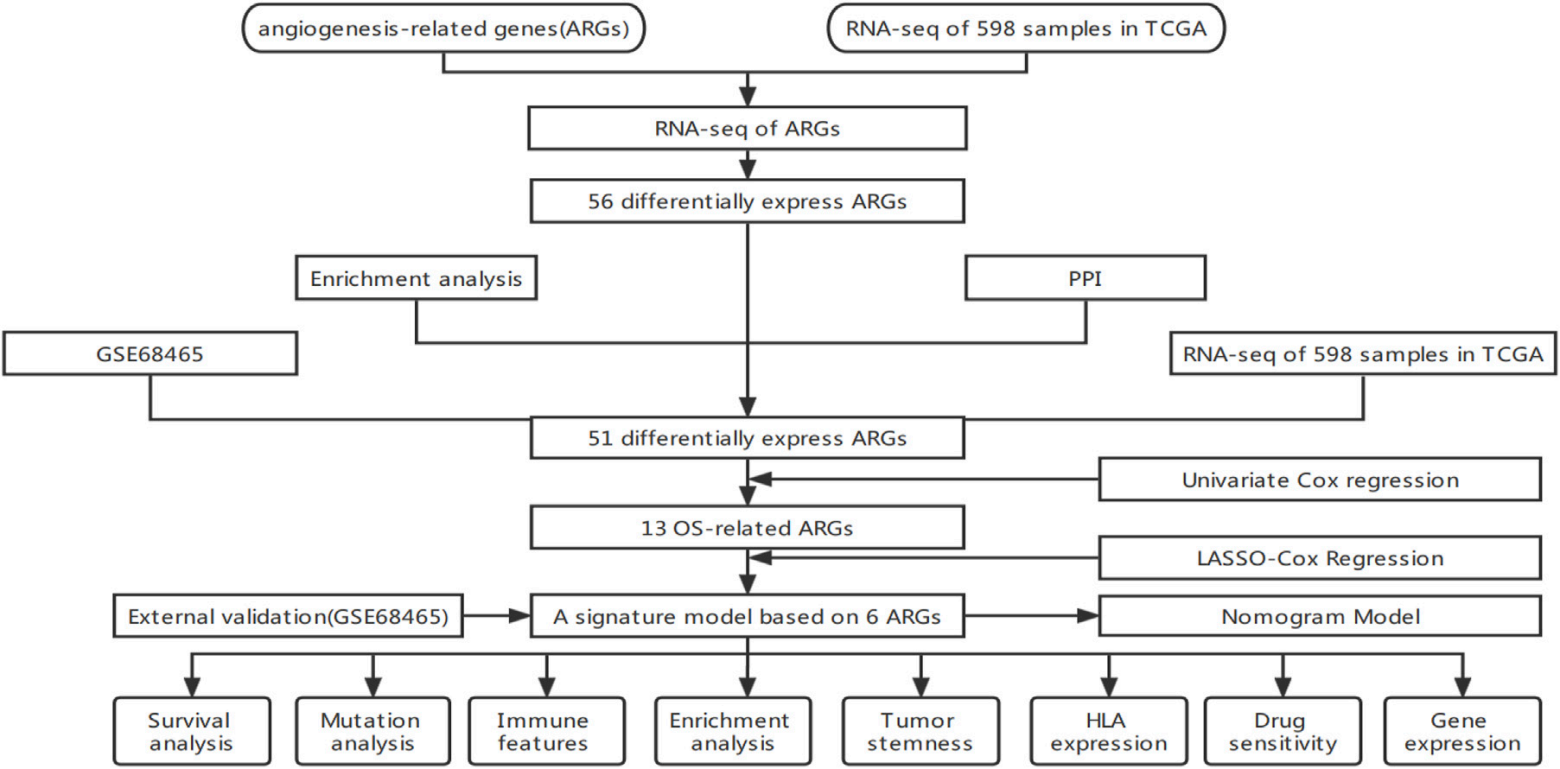
Data gathering and processing flowchart.

This work used TCGA’s comprehensive genome-wide gene expression profile to identify angiogenesis-related genes (AGRs) strongly correlated with lung cancer prognosis. We constructed and validated a diagnostic, prognostic, and recurrence model of lung adenocarcinoma and the corresponding nomogram. Corresponding data from GEO further validate these results. To summarize the flow chart of this study is shown in Figure 1.

## 2 Materials and methods

### 2.1 Selection of angiogenesis-related genes

AGRs were found on the GeneCards website (https://www.genecards.org/) by searching for “angiogenesis.” Correlation scores were used to indicate the strength of the correlation between genes and angiogenic activity, ranging from 0 to 100. High scores indicate strong correlations. For further analysis, we chose ARGs with correlation coefficients more prominent than 5. We then downloaded 36 additional tumor angiogenesis genes (hallmark-angiogenesis (Subramanian et al., 2005)) from Gene Enrichment Analysis (GSEA). By eliminating overlapping genes, a total of 137 angiogenesis-related genes were found.

### 2.2 Acquisition of lung adenocarcinoma datasets

The TCGA database (https://portal.gdc.cancer.gov/) was used to get the mRNA, mutation, and clinical data. There are 594 samples in the mRNA files (535 LUAD and 59 non-tumor samples). GSE68465, GSE50081 and GSEGSE31210 are validation datasets from the Gene Expression Omnibus Database (GEO, https://www.ncbi.nlm.nih.gov/geo/).

### 2.3 Differentially expressed gene screening and gene mutation analysis

To find angiogenic genes associated with LUAD, RNA sequencing data from the TCGA database was matched to a total of 137 ARGs. We then used the R package “limma” to identify differentially expressed genes (DEGs) with an absolute fold change (|logFC|) > 1 and an adjusted *p*-value <0.05. Using the “pheatmap” package, a heatmap was made to visualize differentially expressed genes. The protein-protein interaction (PPI) network is available on the STRING website (http://string.embl.de/) (Szklarczyk et al., 2017).

### 2.4 Functional enrichment analysis

Using the “clusterProfiler” package, KEGG (Kyoto Encyclopedia of Genes and Genomes) and GO (Gene Ontology) analyses were made to determine which pathways and functions angiogenesis-related genes are enriched in. DO (disease ontology) studies were also conducted using the R package “DOSE” to determine if angiogenesis-related genes are implicated in lung disorders, particularly LUAD (Yu et al., 2015).

### 2.5 Construction and validation of a prognostic signature based on angiogenesis-related genes

On a 7:3 ratio, 503 samples were separated into training and validation sets, with no samples designated as controls. Combining ARGs mRNA expression levels and clinical data, Univariate Cox regression analysis was used to find differentially expressed ARGs with significant prognostic significance. A predictive polygenic model was created using identified genes related to overall survival (OS). A minimum absolute shrinkage and selection operator (LASSO) Cox regression approach was used to construct multivariate models of ARG using the “glmnet” package of R software (Sauerbrei et al., 2007). Only genes with non-zero coefficients were chosen in LASSO regression to construct risk scores (Kidd et al., 2018). The best model was determined by maximizing the performance and using the least number of genes.

Subsequently, a predictive risk score formula was established based on a linear combination of expression levels and weighted regression coefficients obtained by LASSO Cox regression analysis. $Risk\;score\;\left(RS\right)=\overset{n}{\underset{i=1}{\sum }}\left(coefi\;*\;Xi\right).$ The median RS was used as the cutoff value to separate the TCGA LUAD cohort into high-risk (HR) and low-risk (LR) subgroups. Using univariate and multivariate Cox regression analysis, the prognostic significance of the model in patients with LUAD was evaluated. Kaplan drew the survival curve–Meier (KM) method and the difference in survival rate between the HR group and LR group was evaluated by log-rank test. Using the receiver operating characteristic (ROC) curve, the prognostic accuracy of the risk prediction model was evaluated. In addition, the clinical significance of this model was evaluated. These results were then tested in another LUAD cohort in the GEO dataset by survival analysis and ROC curve analysis. Furthermore, the nomogram with calibration plots was built using the rms R package to forecast the concordance between actual and predicted survival.

### 2.6 Tumor mutation burden and gene mutation analysis

Somatic mutations in the TCGA were studied using the R package “maftools” to identify variations in somatic mutations between the HR and LR groups (Mayakonda et al., 2018). Subsequently, we estimated the two groups’ tumor mutation burden (TMB) per patient. Mutations in signature genes were searched on the cBioPortal website (https://www.cbioportal.org).

### 2.7 Immune microenvironment analysis

Immune cell infiltration was identified using timer 2.0 (cistrome.shinyapps.io/timer/) *via* the MCPCOUNTER, CIBERSORT, QUANTISEQ, Timer, CIBERSORT-ABS, EPIC, and XCELL algorithms. Infiltration levels of stromal and immune cells can be calculated with the ESTIMATE algorithm (Yoshihara et al., 2013). The enrichment score of genes in a particular gene set can be calculated by ssGSEA (single-sample gene set enrichment analysis). The process of ssGSEA includes ranking genes according to the absolute expression of genes in the sample and then calculating the enrichment score by integrating the differences between the empirical cumulative distribution functions of gene ranking (Bindea et al., 2013; Finotello and Trajanoski, 2018). Concentration scores of 16 immune cells were calculated using “GSEABase” and “GSVA” packages. The TIMER database studied six immune cells (B cells, macrophages, neutrophils, dendritic cells, CD8^+^ T cells, and CD4^+^ T cells) infiltration for its association with gene expression. Gene copy number variation in the TIMER database was studied for its potential impact on immune cell infiltration.

The expression of multiple immune checkpoint molecules was compared to determine whether there were differences in immune checkpoint blockade (ICB) therapy between the HR and LR groups. Immune checkpoints with differential expression between the two groups were visualized. Additionally, TIDE (Tumor Immune Dysfunction and Exclusion) score was calculated online following the instructions (https://tide.dfci.harvard.edu/). An inverse correlation was found between the TIDE score and ICB treatment success (Jiang et al., 2018).

### 2.8 Antigen presentation analysis

Human leukocyte antigen (HLA), found on numerous immune cells’ surfaces, is crucial for triggering cellular and humoral immunity (Halpert et al., 2020). To determine whether or not there were distinctions in antigen expression between the two groups, the “limma” package was used to compare the HLA expression levels of the two groups.

### 2.9 Cancer stem cell infiltration analysis

The UCSC Xena browser (http://xena.ucsc.edu/) was used to extract the DNA methylation-based stemness scores (DNAss) and RNA-based stemness scores (RNAss) of TCGA-LUAD patients. We conducted a comparative study at the DNA and RNA levels to examine the variations in stem cell infiltration between the two groups.

### 2.10 Gene set enrichment analysis (GSEA)

The c2. cp.kegg.v7.5.1. symbols and c5. go.v7.5.1. symbols collection was used to explore the function annotation in HR and LR groups using the R package “org. Hs. eg. db”. Gene sets with FDR <0.05 were considered statistically significant.

### 2.11 Predicting drug therapeutic response

Using the Cancer Immunome Atlas (https://tcia.at/), immunophenoscore (IPS) was derived to predict the sensitivity to immunotherapy. The IC50 of common chemotherapeutic agents in the total TCGA cohort was calculated by the administered “pRRophetic” software package to assess the predictive ability of AGRs for drug treatment response. The IC50 differences between the HR and LR groups were then compared using the Wilcoxon ranking test. Finally, we applied the “ggplot” R package to draw the results into bar charts.

### 2.12 Verification of signature genes in databases

The Cancer Cell Line Encyclopedia (CCLE) Line (http://www.broadinstitute.org/ccle) provides downloadable information on the mRNA expression levels of several LUAD cell lines. The Human Protein Atlas (HPA) database was used to verify protein expression levels.

### 2.13 Quantitative real-time PCR (qPCR)

The expression of model genes in humans was confirmed using a quantitative real-time PCR technique. Total RNA was isolated from human tissues initially using the Trizol reagent. Then, reverse transcription was used to transform the isolated RNA into cDNAs. Finally, the quantitative real-time PCR technique was used to assess the expression levels of model genes in human tissues. The primers are shown in Supplementary Table S3. Relative expression values of model genes were calculated using the 2^−ΔΔCT^ method and normalized with beta-actin. The experiments were reviewed and approved by the authority (Ethics No: WDRY2022-K041) and executed in accordance with relevant guidelines.

### 2.14 Statistical analysis

R was used for all statistical analysis and graphical creation (version 4.2.1). Volcano plots were drawn with the “ggplot2″ package. Violin diagrams are drawn with “ggpubr” packets. The Mann-Whitney test was utilized for differentially expressed gene analysis, tumor mutation burden analysis, ssGSEA score, immunological checkpoint analysis, and HLA analysis. Cancer stem cell infiltration and drug sensitivity tests were conducted using a correlation test. The log-rank test and Kaplan-Meier analysis compared overall survival (OS) between groups.

## 3 Results

### 3.1 Identification and exploration of angiogenesis-related differentially expressed genes

**FIGURE 2:**
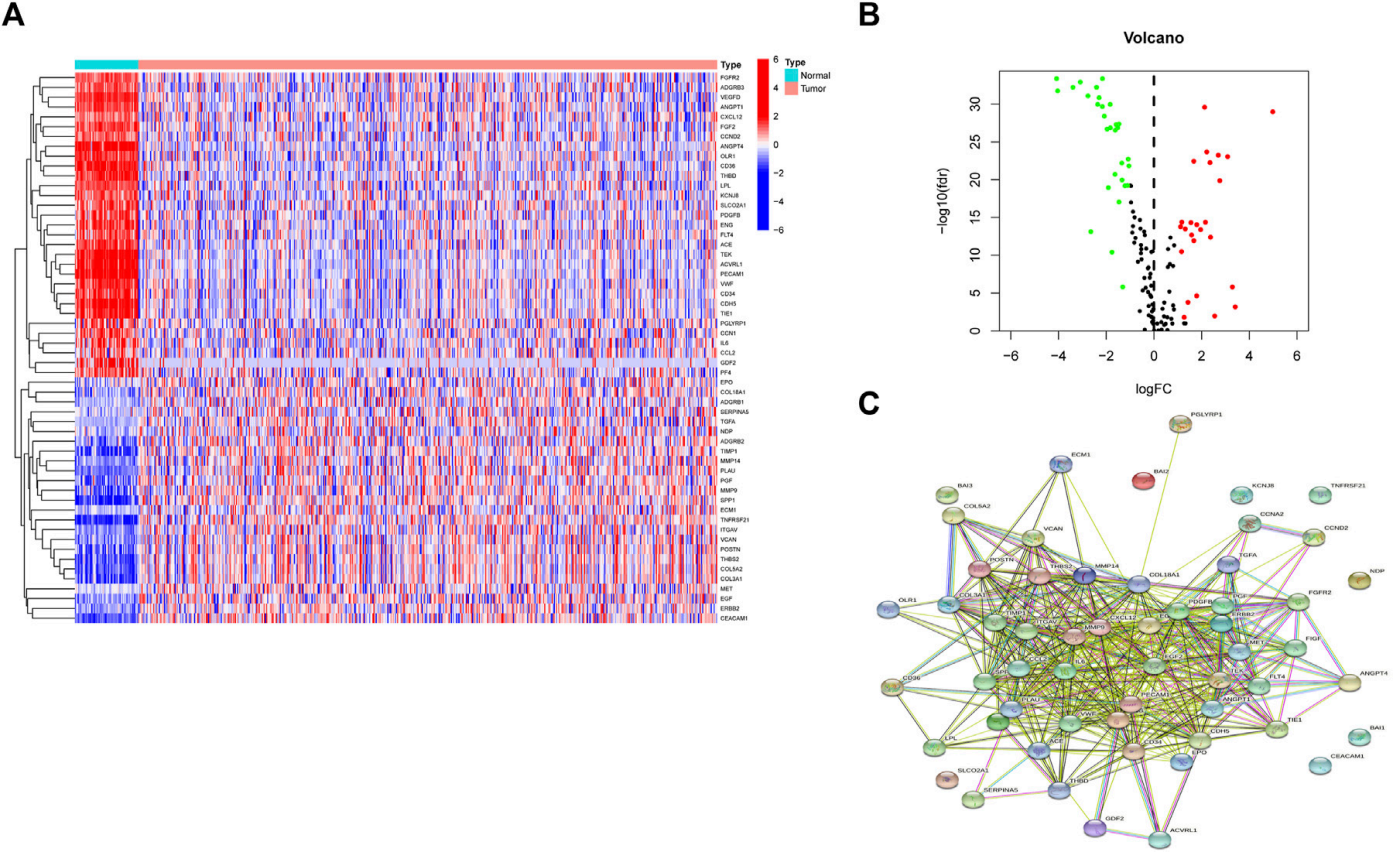
A sum of 56 genes showed significant differential expression. **(A)** Heatmaps of differentially expressed angiogenesis-related genes. **(B)** Volcano plots of differentially expressed angiogenesis-related genes. **(C)** PPI of 56 genes.

**FIGURE 3:**
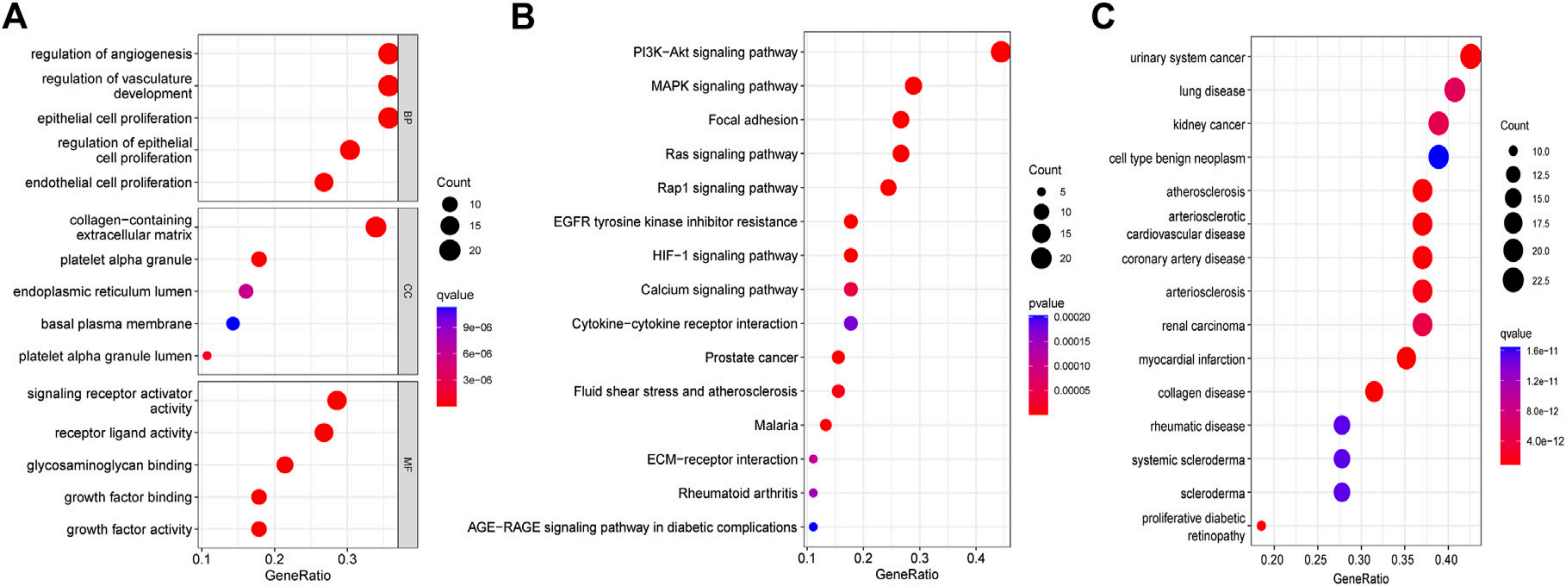
Functional enrichment analysis of differential genes. **(A)** GO. **(B)** KEGG. **(C)**DO.

Differential expression analysis found fifty-six genes to be expressed differently between 539 cancers and 59 normal samples (Figure 2A). Twenty-five genes were elevated, and 31 were downregulated in tumor samples relative to normal samples (Figure 2B). PPI revealed that the majority of genes are linked (Figure 2C). According GO enrichment analyses, different ARGs are crucial for LUAD angiogenesis and vascular growth (Figure 3A). KEGG pathway enrichment analysis revealed that differentially expressed ARGs were primarily implicated in tumor angiogenesis pathways such as PI3K-Akt, MAPK, and Rap1 (Figure 3B). DO research revealed that they were linked to lung illness, lung adenocarcinoma, non-small cell lung cancer, and other conditions (Figure 3C, Supplementary Table S1).

### 3.2 Developing an RS prediction model for angiogenesis-related genes

**FIGURE 4:**
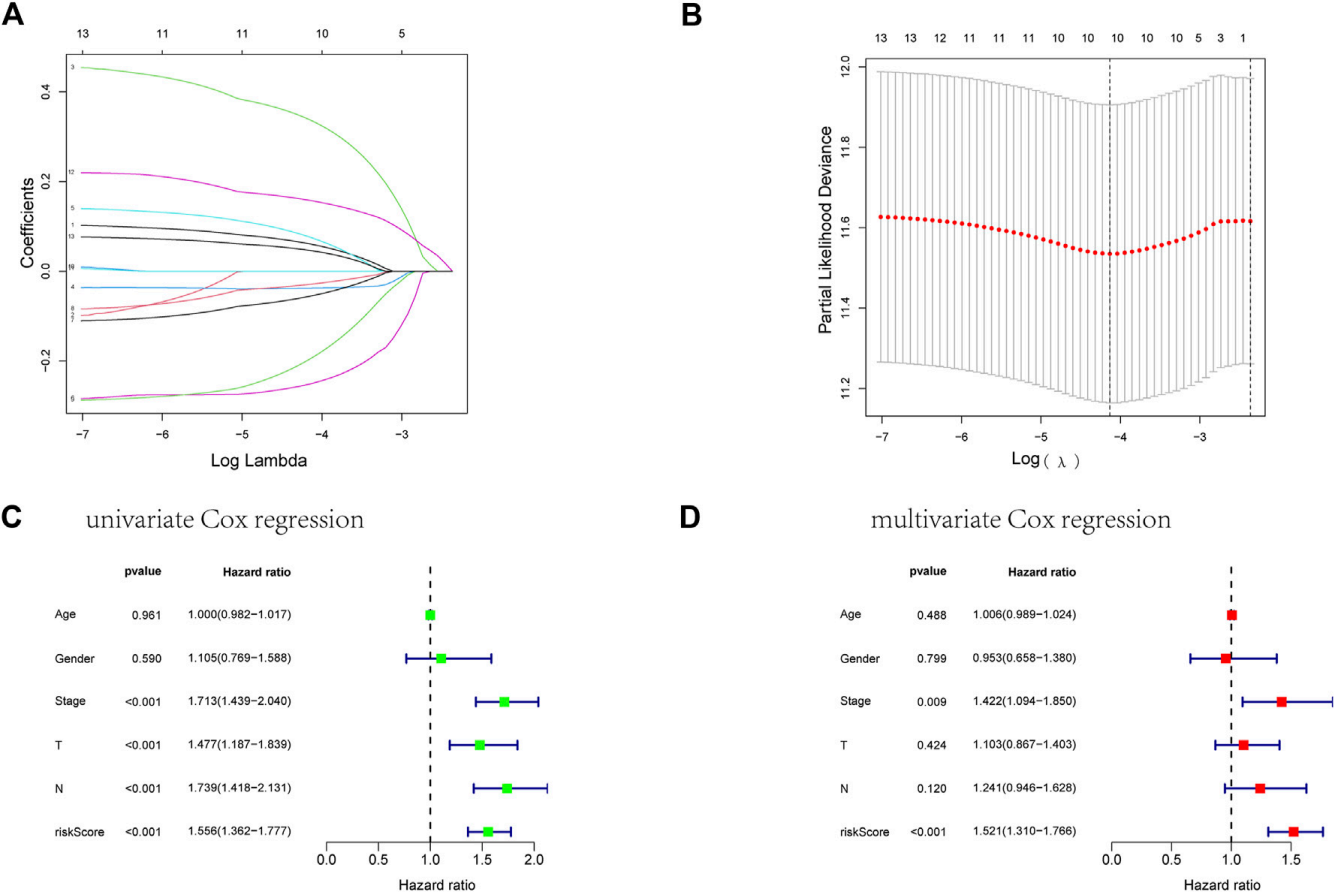
Establishment of an ARGs prognostic model about LUAD’s prognosis using a lasso regression model. **(A)** A LASSO coefficient profile of the 13 ARGs. **(B)** A coefficient profile plot was generated against the log(lambda) sequence. **(C)** Univariate COX regression analysis for RS in TCGA training cohort patients with LUAD. **(D)** Multivariate Cox regression analysis for RS in TCGA training cohort patients with LUAD.

**FIGURE 5:**
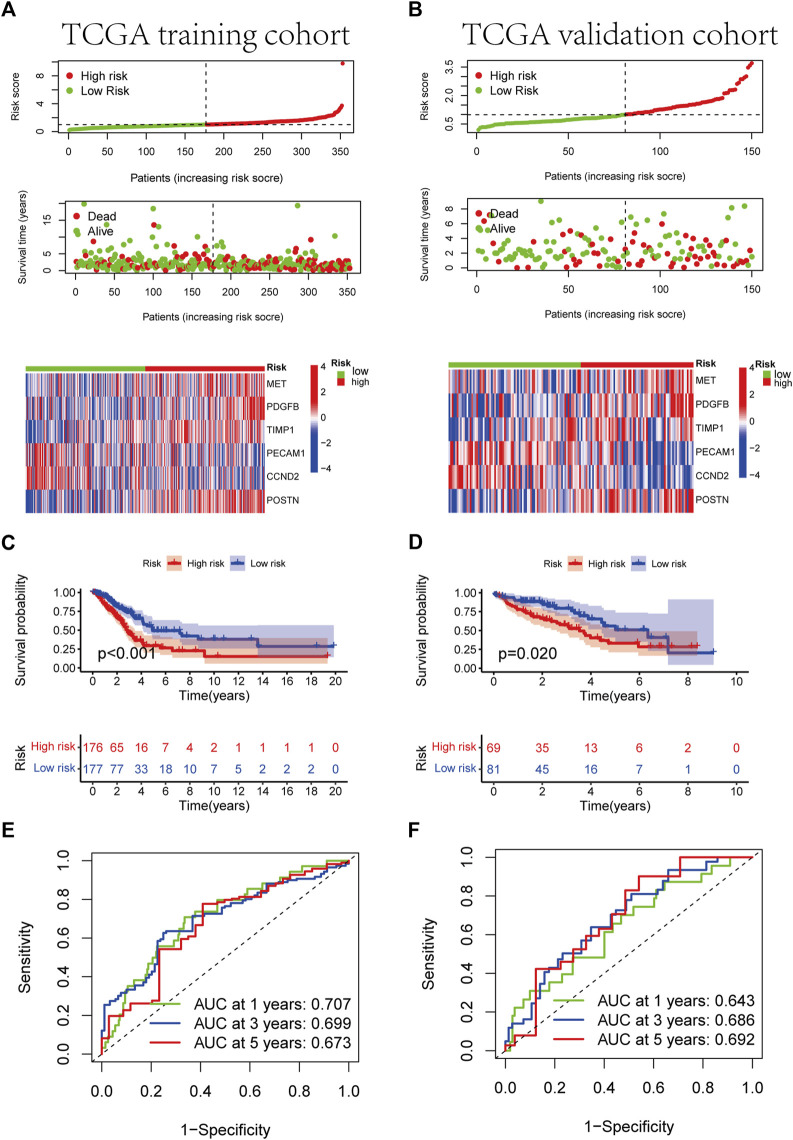
Development of RS based on the 6 ARGs signature of patients with LUAD in the TCGA training and TCGA validation cohorts. **(A, B)** The heatmap of the 6 ARGs expression profiles between the high-risk and low-risk groups in the training or validation cohort, together with the RS distribution and patient vital status. **(C, D)** Kaplan-Meier analysis of the prognostic model in training or validation cohort. **(E, F)** The ideal AUC of the gene signature in the two cohorts was determined using a time-dependent ROC analysis.

**FIGURE 6:**
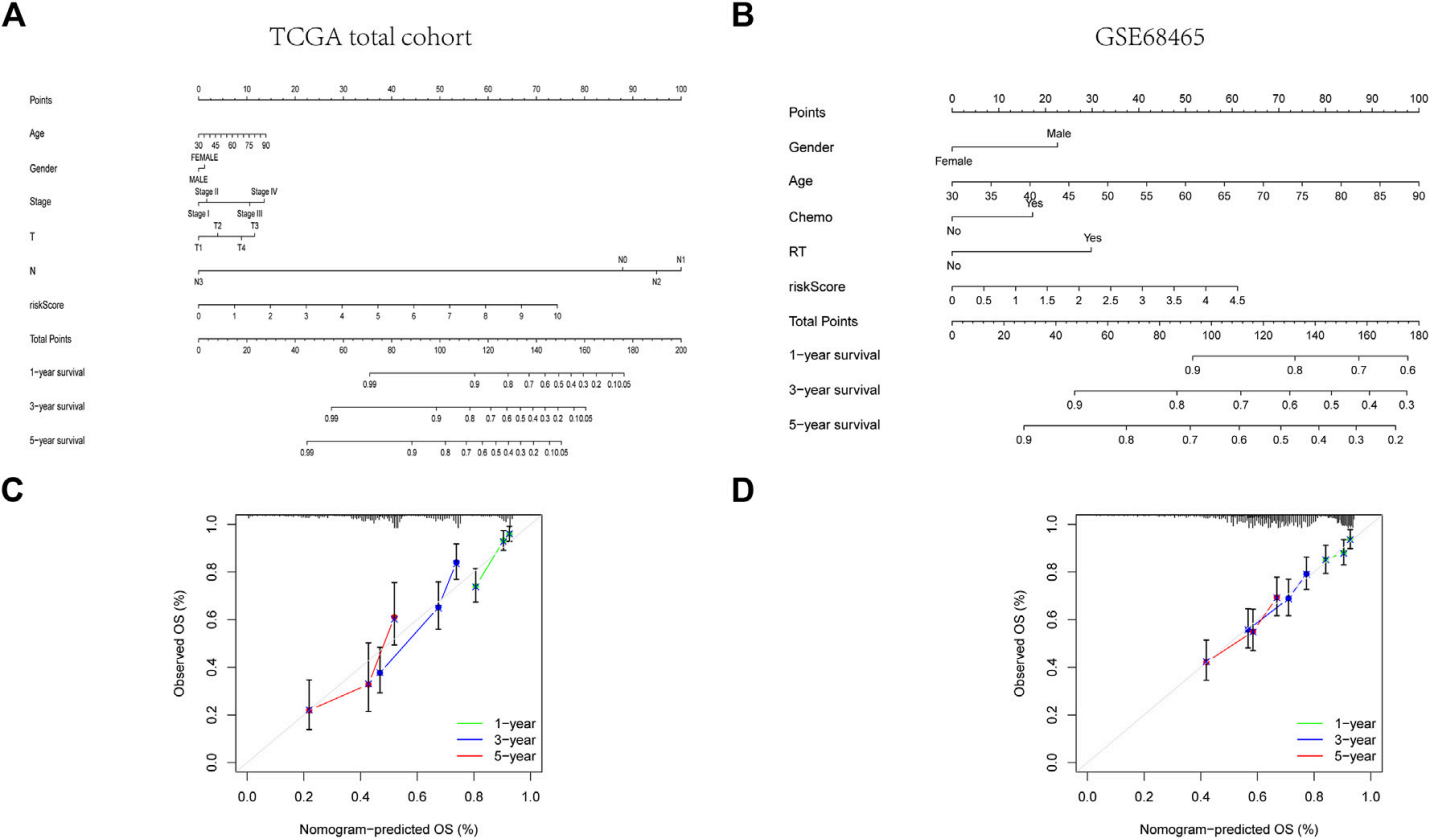
Construction of a predictive nomogram. **(A, B)** The nomogram for OS prediction in LUAD patients at 1, 3, and 5 years. **(C, D)** Calibration curves of the nomogram for OS prediction at 1, 3, and 5 years.

**FIGURE 7:**
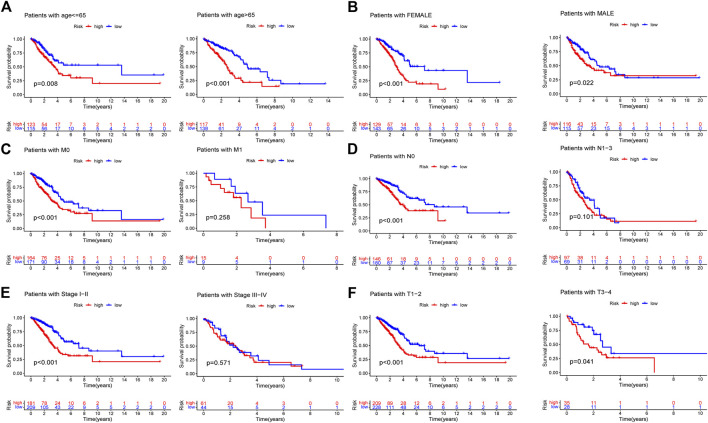
Subgroup study of various clinical characteristics using the RS formula. **(A)** Age>65 years and age≤65 years. **(B)** FEMALE and MALE. **(C)** M0 and M1. **(D)** NO and N1-3. **(E)** Stage I-II and stage III-IV. **(F)** T1-2 and T 3-4.

First, we took the intersection of the total TCGA cohort with GSE68465 to obtain the common genes. Subsequently, the two cohorts intersected with differentially expressed genes, and 51 genes were obtained for subsequent analysis. Thirteen of the 51 differentially expressed genes correlate with the overall survival of LUAD patients in the TCGA cohort (Supplementary Figures S1A–N). Then ten genes in these 13 genes were finally screened by Lasso regression (Figures 4A, B). Finally, the multivariate cox regression revealed six of these ten angiogenic-associated genes (MET, PDGFB, TIMP1, PECAM1, CCND2, POSTN) as independent risk variables to build risk models, the angiogenesis-related genes (ARGs) risk model. And the ARGs risk score formula was as follows: RS=0.0996201384411945*MET+0.416174488743696* $PDGFB+0.180348206385705*TIMP1+\left(-0.398844301036605*PECAM1\right)+$ $\left(-309425270158878*CCND2\right)+0.202128532193696*POSTN$ Four patient cohorts’ risk scores were calculated, and the patients were then split into high-and low-risk groups based on the median risk score for each cohort. In the discovery cohort (*p* < 0.001), validation cohort (*p* = 0.02), total TCGA cohort (*p* < 0.001), and GSE68465 cohort (*p* = 0.021), GSE31210 cohort (*p* = 0.003), GSE50081 cohort (*p* = 0.038), patients in the high-risk category had inferior outcomes (Figures 5C, D, Supplementary Figures S2C, D and Supplementary Figures S5C, D). Significant differences were between the HR and LR groups in terms of the distribution of RS, the health state of the patients, and the heatmap of the expression profiles of the nine ARGs (Figures 5A, B, Supplementary Figures S2A, B, and Supplementary Figures S5A, B). In addition, risk scores for ARGs were demonstrated to be independent predictive indicators for LUAD patients in all four cohorts (Figures 4C, D and Supplementary Figures S3A–C). To predict overall survival in LUAD patients, we created a nomogram based on patient age, TNM stage, and the ARGs risk score (Figures 6A, B). In addition, the ROC curves of 1-,3-, and 5-year OS demonstrated that our model has a high predictive capacity (Figures 5E, F and Supplementary Figures S2E, F). Calibration plots demonstrated a remarkable consistency between the predicted and observed outcomes (Figures 6C, D). Patients in the entire TCGA cohort were subsequently stratified based on clinical traits in determining the relationship between RS and OS in LUAD patients. The findings revealed that RS strongly predicts outcomes in LUAD patients with various clinical characteristics, particularly in early LUAD patients (Figures 7A–F). In order to demonstrate the predictive performance of our model, we compared it with other models based on the TCGA-LUAD database, such as the published gene prediction models of Duan et al. (2021), Zhang et al. (2022), Yang et al. (2022), Xu and Chen, (2021), Gong et al. (2022). Our model has the greatest c-index of nomogram compared to previously published lung cancer models, as indicated by the findings. These results indicate that our approach is superior to other models in predicting patient prognosis (Supplementary Table S4). These findings imply that a risk model based on six genes predicts OS in individuals with LUAD.

### 3.3 Tumor mutation burden and gene mutation analysis

**FIGURE 8:**
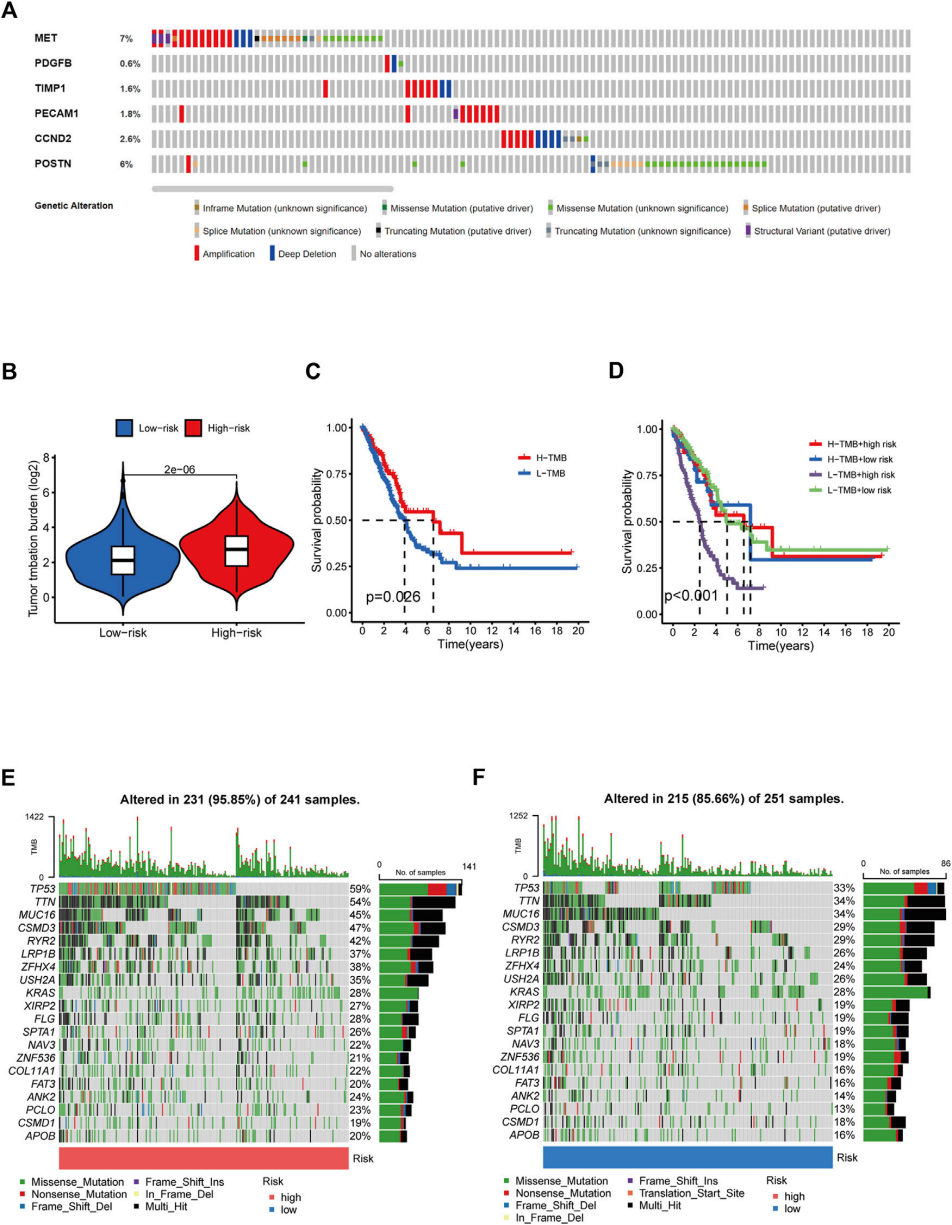
Tumor mutation burden and gene mutation analysis. **(A)** Genetic alteration analysis of the ARGs in the TCGA LUAD cohort. **(B)** TMB between the low- and high-risk subgroups based on RS. **(C)** Survival study of the various TMB stratification groups. **(D)** Analysis of the survival of various groups stratified by TMB and RS. **(E, F)** Assessment of the differences in the mutational landscape between high- and low-risk.

Given that TMB is linked to immunotherapy effectiveness, we calculated the difference in TMB value between the two groups. TMB was considerably more remarkable in the HR group than in the LR group (Figure 8B). However, those with elevated TMB had a favorable survival advantage (Figure 8C). The utility of integrating risk scores with TMB to predict patient outcomes was then investigated. The Kaplan-Meier analysis suggested that LR scores and high TMB were associated with more prolonged survival (Figure 8D). We analyzed mutation rates between the HR and LR groups. The findings revealed a higher frequency of mutational events within the HR group. The most prevalent alterations in the HR group, TP53 and TTN were also considerably more frequent than those in the LR group. The other six LUAD mutated genes (MUC16, CSMD3, RYR2, LRP1B, ZFHX4, and USH2A) also showed increasing trends to different degrees (Figures 8E, F). Using the LUAD cohort from the cBioportal database, we analyzed the value of six angiogenic genes (MET, PDGFB, TIMP1, PECAM1, CCND2, POSTN) in the development of diagnostic, prognostic, and recurrence models. The study found that 19.6% (566 LUAD samples) showed genetic alterations, of which 7% occurred in MET, whose primary alteration was amplification, and 6% of mutations occurred in POSTN (Figure 8A). These genes may be used as therapeutic targets, offering fresh avenues for treating LUAD.

### 3.4 Immune features analysis

**FIGURE 9:**
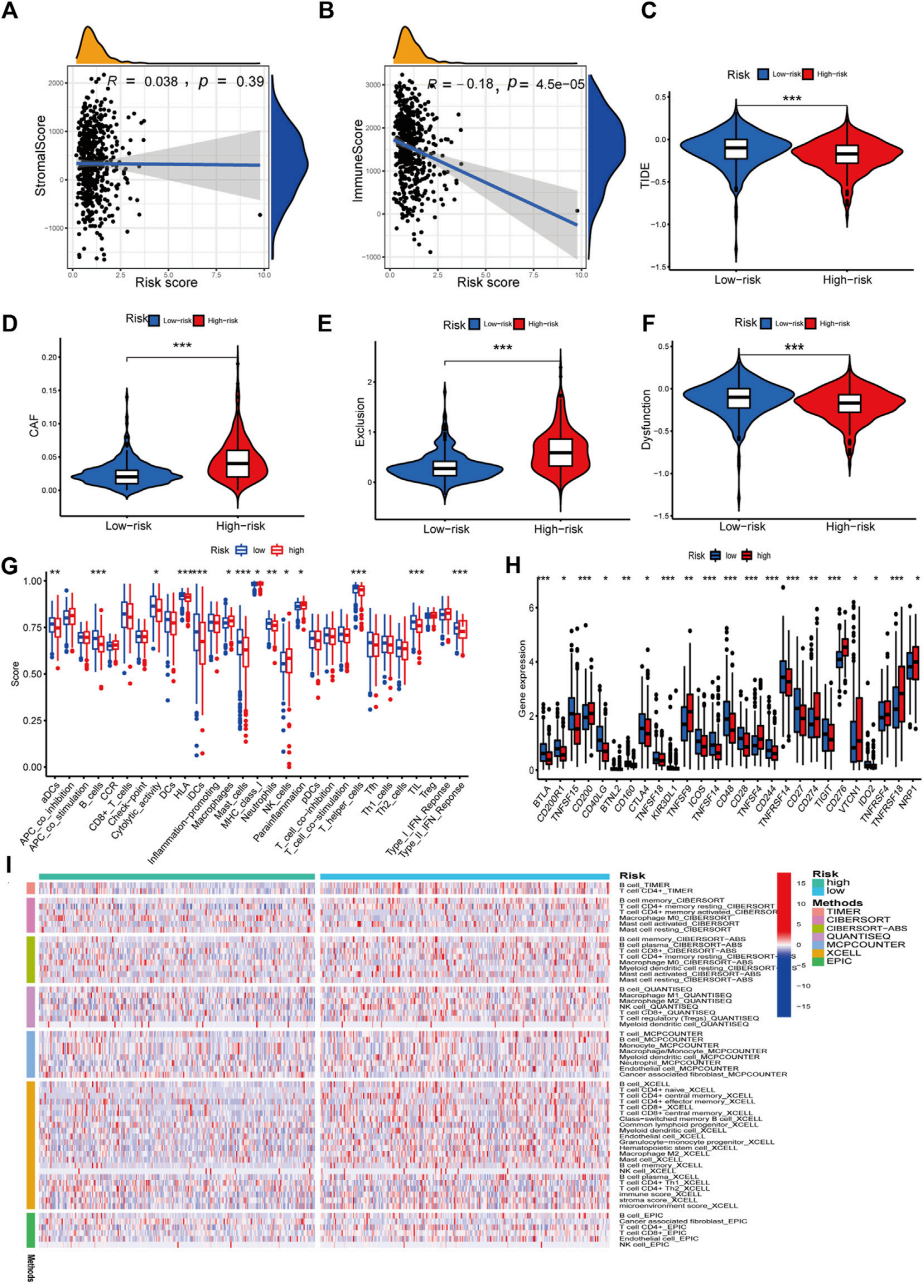
Immune features analysis. **(A)** Correlation analysis of the risk score and immune score. **(B)** Correlation analysis of the risk score and stromal score. **(C)** Comparison of TIDE and risk score. **(D)** Comparison of CAF and risk score. **(E)** Comparison of T-cell exclusion and risk score. **(F)** Comparison of T-cell dysfunction and risk score. **(G)** Infiltration score of immune cells. **(H)** Expression of immune checkpoints. **(I)** Heatmap of immune response in high and low-risk groups based on multiple algorithms. *p < 0.05, **p < 0.01, ***p < 0.001.

We investigated the relationship between RS and the immune status of patients in the TCGA cohort and found a significant change in immune cells. From the results, the LR group had a better immune status (Figure 9I). While there was a negative correlation between the ESTIMATE score of immune cells and the risk score, there was a positive correlation between the ESTIMATE score of stromal cells and the risk score (Figures 9A, B). Next, we compared the immune cell scores of the two patient groups. The total TCGA cohort showed that the LR group’s infiltration scores of most immune cells were higher than in HR groups, such as aDC, B cells, and T-helper cells (Figure 9G). Considering the importance of checkpoint inhibitors in clinical treatment, we further analyzed the differences in ICBs expression and found substantial differences in CTLA4, CD28, ID02, and CD27 between the two groups (Figure 9H). We next evaluated the potential therapeutic effectiveness of immunotherapy in both patient groups using TIDE. A higher TIDE prediction score, the higher the likelihood of immune evasion, suggesting that patients are less likely to benefit from ICI therapy. We found that patients in the HR group had a lower TIDE score than those in the LR group, suggesting that the HR patients might respond better to ICI therapy (Figure 9C). In addition, we discovered that CAF and T-cell exclusion ratings were more excellent in the HR group, whereas T-cell dysfunction scores were higher in the LR group (Figures 9D–F).

**FIGURE 10:**
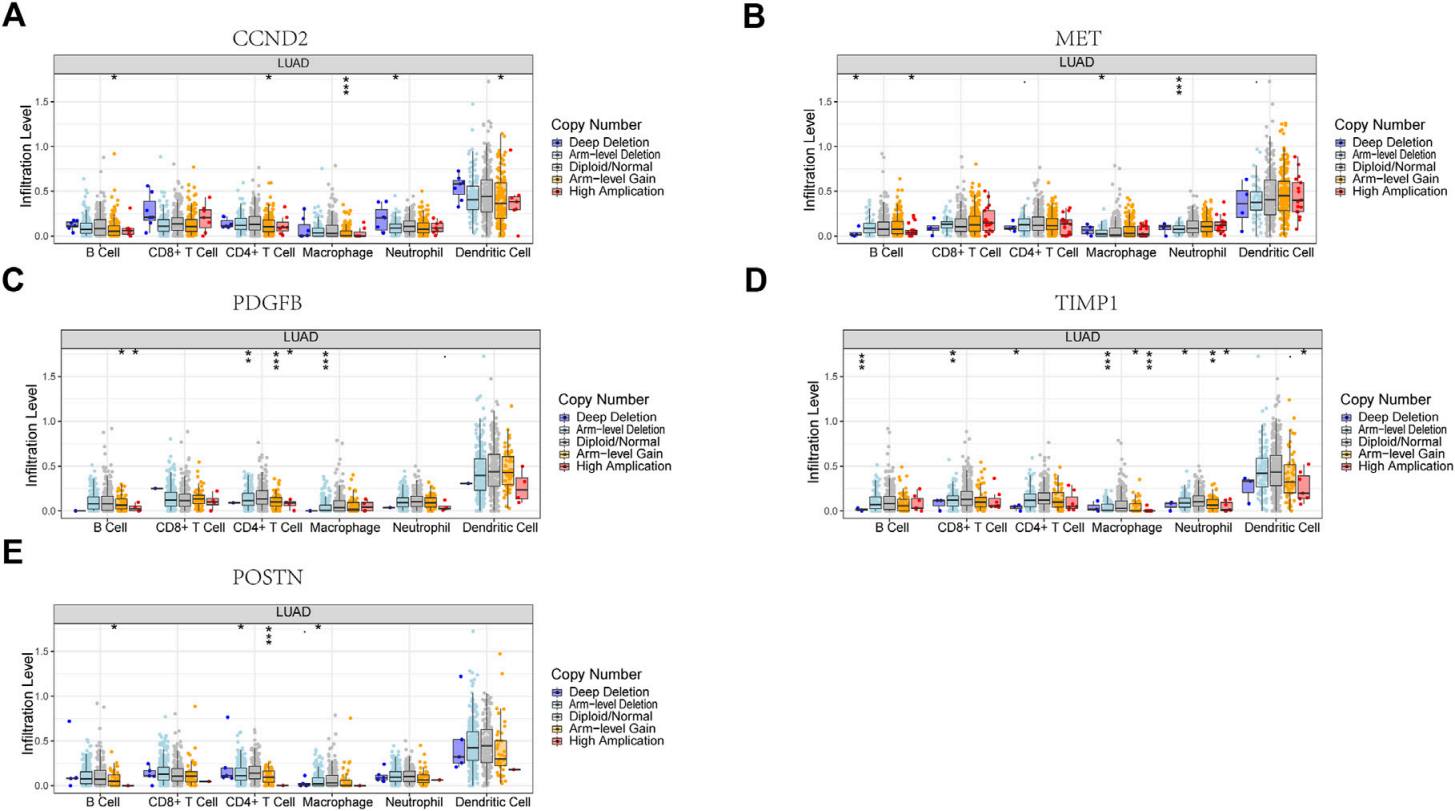
Association between CNV and immunity in 5 model genes. **(A)** CCND2. **(B)** MET. **(C)** PDGFB. **(D)** TIMP1. **(E)** POSTN. *p < 0.05, **p < 0.01, ***p < 0.001.

Six signature genes were revealed to be linked with immune cell infiltration. Positive correlations were seen between MET and CD4^+^ T cells, macrophages, dendritic cells, CD8^+^ T cells, and neutrophils, but not B cells (Supplementary Figure S4B). PDGFB was associated favorably with the other four immune cells and negatively with B cells, but not with CD8^+^ T cells (Supplementary Figure S4C). POSTN was negatively connected to B cells and favorably related to the other four immune cells, but not to CD4^+^ T cells (Supplementary Figure S4E). Positive correlations were seen between TIMP1 and CD4^+^ T cells, macrophages, dendritic cells, and neutrophils, but not CD8^+^ T cells and B cells (Supplementary Figure S4F). Positive correlations were seen between CCND2 and PECAM1 and all six immune cells (Supplementary Figures S4A, D). Copy number variation in genes may affect immune cell infiltration compared to the diploid/normal group. As PECAM1 was not available in the database, we analyzed the copy number variation of the remaining 5 cell types about immune cells. MET copy number variants were significantly associated with decreased levels of three immune cell types (B cells, neutrophils, and macrophages), POSTN copy number variants were significantly associated with decreased levels of three immune cell types (B cells, CD4 + T cells, and macrophages), TIMP1 copy number variants were significantly associated with decreased levels of six immune cell types (B cells, CD4 + T cells, CD8 + T cells, neutrophils, CD4^+^ T cells, CD8^+^ T cells, neutrophils, macrophages, and dendritic cells), PDGFB copy number variation was significantly associated with decreased levels of three immune cells (B cells, CD4^+^ T cells, and macrophages), and CCND2 copy number variation was significantly associated with decreased levels of five immune cells (B cells, CD4^+^ T cells, neutrophils, macrophages, and dendritic cells) (Figures 10A–E). These results suggest that changes in model gene copy number can significantly modulate immune cell infiltration.

### 3.5 Cancer stem cell infiltration analysis

**FIGURE 11:**
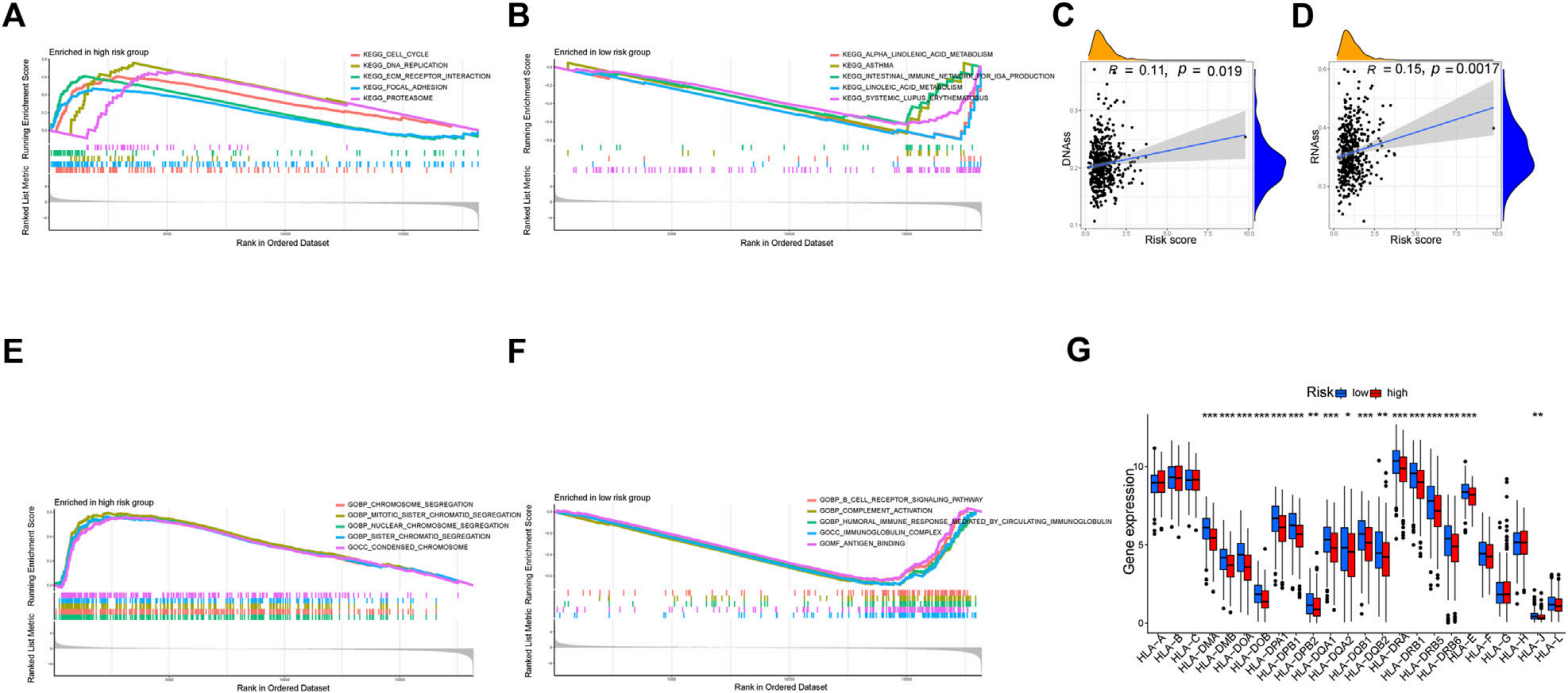
Exploration of possible reasons for differences between high and low-risk groups. **(A, B)** KEGG Enrichment Analysis. **(C, D)** Tumor stem cell analysis. **(E, F)** GO Enrichment Analysis. **(G)** Expression of HLA. *p < 0.05, **p < 0.01, ***p < 0.001.

We used DNAss and RNAss to compare tumor stemness amongst distinct risk patterns. The results demonstrated a positive relationship between risk scores and DNAss (r = 0.11, *p* = 0.019) and RNAss (r = 0.15, *p* = 0.0017) values (Figures 11C, D). This shows that the HR group’s LUAD cells exhibited a more marked stem cell profile and a lower degree of cell differentiation.

### 3.6 Gene set enrichment analyses

According to KEGG, pathways enriched in the HR group included the cell cycle, DNA replication, proteasome, and focal adhesion, whereas pathways enriched in the LR group included asthma, linoleic acid metabolism, systemic lupus erythematosus, and alpha-linolenic acid metabolism (Figures 11A, B). Additionally, GO revealed that genes in the LR group were engaged in complement activation, antigen binding, immunoglobulin complexes, and other processes, whereas genes in the HR group were enriched in chromosome segregation, condensed chromosomes, and nuclear chromosome segregation (Figures 11E, F).

### 3.7 Antigen presentation analysis

There was a substantial variation in HLA expression associated with antigen presentation between the HR and LR groups. The expression of numerous HLA classes I and II was more significant in the LR group than in the HR group in the total TCGA cohort (Figure 11G).

### 3.8 Drug sensitivity analysis

**FIGURE 12:**
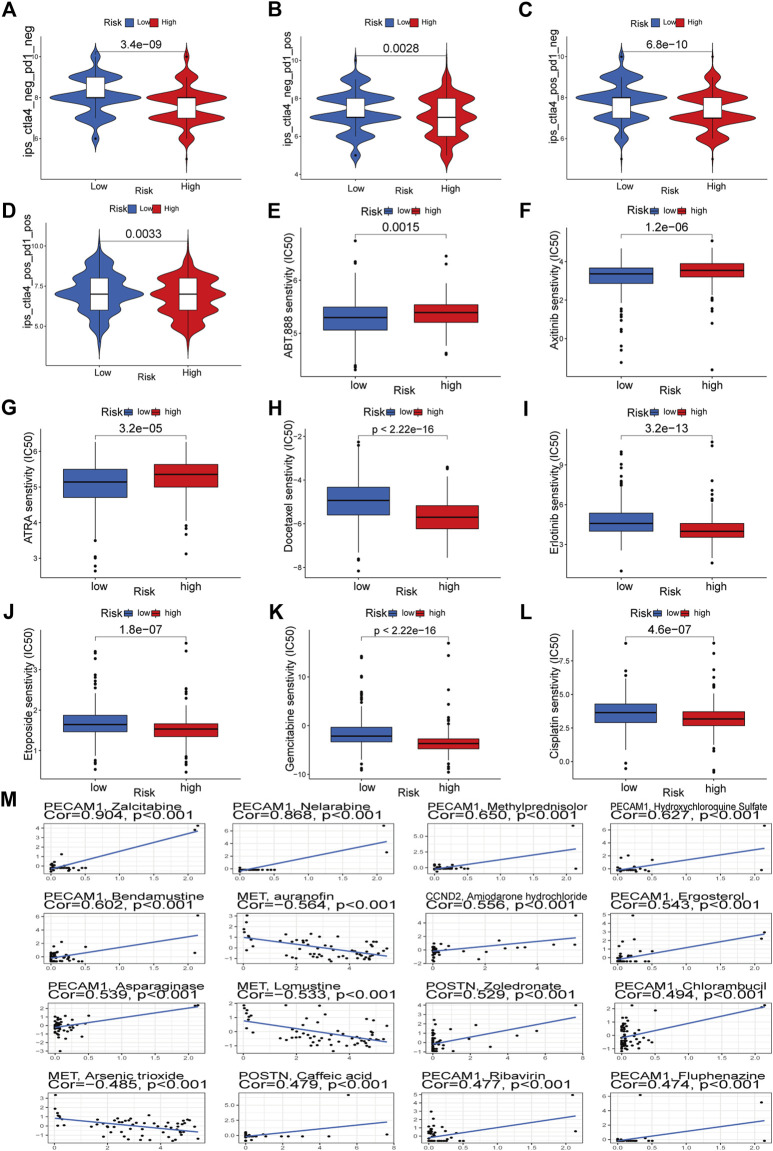
Immunotherapeutic responses of the ARGs prognostic signature and drug sensitivity analysis. **(A–D)** Comparison of the immunophenoscore (IPS) between the low- and high-risk groups stratified by both CTLA4 and PD-1. **(E–L)** Drug sensitivity analysis. **(M)** Multiple drugs for six signature genes.

Through TCIA, we specifically looked at the impact of risk scores on the efficacy of immunotherapy. The results demonstrated that the HR group was more likely to respond to CTLA4-positive/PD-L marker-positive treatment than the LR group (Figures 12C, D). This shows that patients in the HR group may respond better to CTLA4-positive/PD-L marker-positive immunotherapy, leading to a better clinical outcome. Patients in the HR group were more responsive to docetaxel, erlotinib, etoposide, gemcitabine, and cisplatin, as evidenced by their lower IC50s compared to those in the LR group (Figures 12H–L). The IC50 of ABT.888 (also known as veliparib), axitinib, and ATRA was higher in the HR group than in the LR group (Figures 12E–G). We examined the association between six signature genes and medication effectiveness in NCI-60 cell lines, including the LUAD cell line, to guide gene-targeted treatment. The findings of the first sixteen studies were shown based on the order of the *p*-values, from smallest to most lavish. PECAM1 was sensitive to bendamustine and methylprednisolone, and zalcitabine, MET was sensitive to auranofin, lomustine, and arsenic trioxide, and CCND2 was sensitive to amiodarone hydrochloride (Figure 12M). POSTN was sensitive to zoledronic acid and caffeic acid (Figure 12M). Supplementary Table S2 also lists the names of the medicines and genes along with their connection coefficients.

### 3.9 Verification of signature genes

**FIGURE 13:**
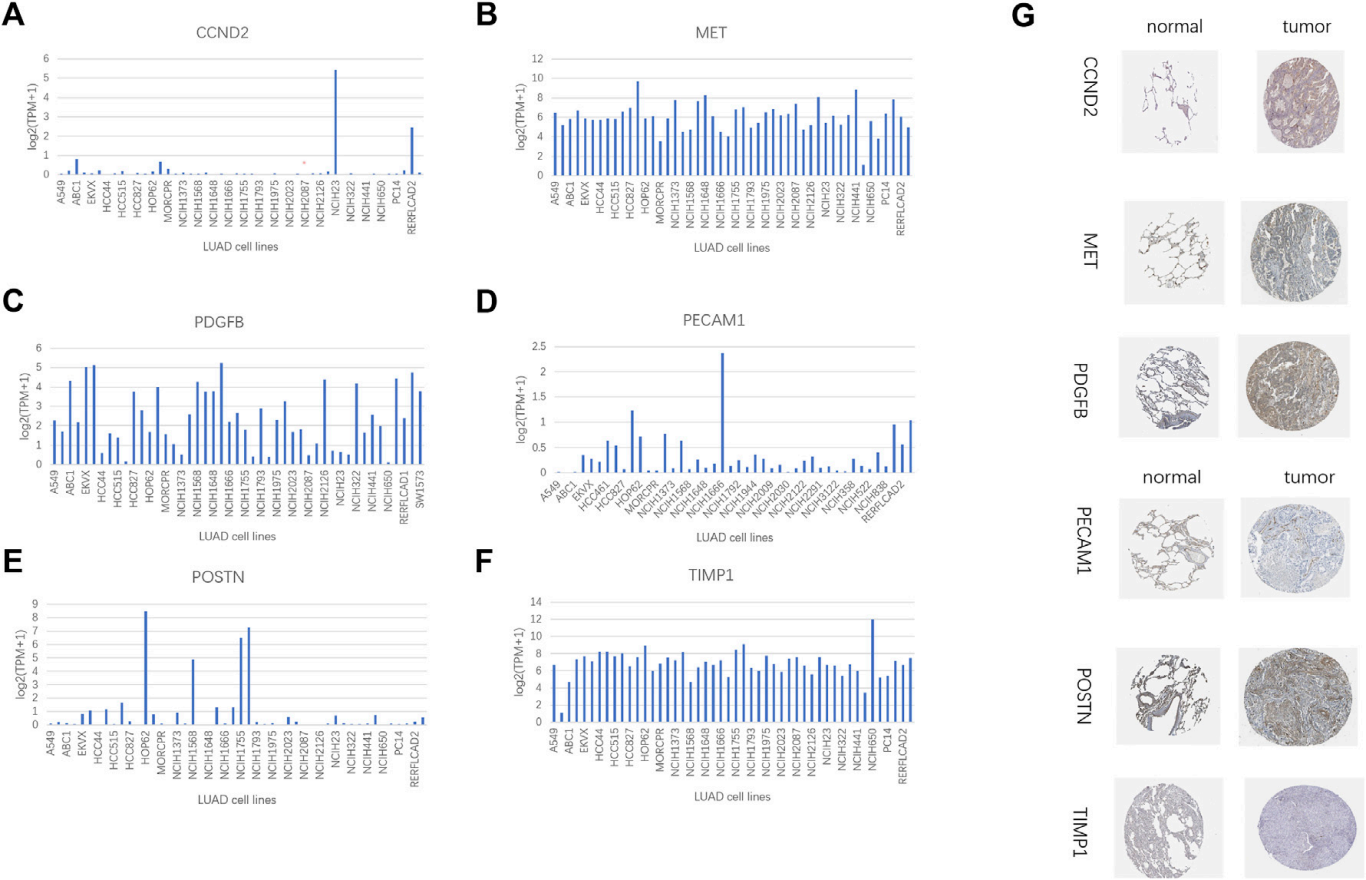
HPA database and compare gene expression in LUAD cell lines. **(A)** CCND2. **(B)** MET. **(C)** PDGFB. **(D)**PECAM1. **(E)** POSTN. **(F)** TIMP1. **(G)** Immunohistochemical results of 6 model genes in the HPA database.

The IHC data for six proteins in the HPA database were evaluated. Despite the inability to precisely assess the differences between normal lung tissue and malignancies, the preliminary findings suggest increased expression of CCND2 and PDGFB in tumor tissues (Figure 13G). Meanwhile, PECAM1 and POSTN levels may be lowered in tumorous tissues (Figure 13G). There was no discernible variation in the levels of MET and TIMP1 expression (Figure 13G). In several LUAD cell lines, the same gene is expressed differently (Figures 13A–F). We employed a quantitative real-time PCR technique to measure the expression levels of model genes in human tissues to confirm our prognostic model’s validity further. The findings revealed that MET, PDGFB, PECAM1, POSTN, and TIMP1 expression differed in human tissues and were consistent with the TCGA database (Supplementary Figures S6A–E). In human tissues, however, there was no variation in CCND2 expression (Supplementary Figure S6F).

## 4 Discussion

The vast majority of cases of NSCLC are LUAD (Bade and Dela Cruz, 2020). LUAD has a poor prognosis due to a lack of knowledge of its pathogenesis (Peng et al., 2021). Targeted treatment and immunotherapy for LUAD have made significant strides in recent years due to researchers examining the etiology of LUAD from various routes and biological processes. Despite this progress, it remains difficult for doctors to assess the risk of LUAD and make prognostic predictions for their patients. Since the numerous scoring methods now used in clinical practice, such as the TNM staging system, have more or fewer limitations, there is an urgent need for novel molecular biomarkers to predict the survival of LUAD patients. Angiogenesis plays a crucial role in tumor formation; hence, we developed and validated a risk model to predict the prognosis of LUAD patients based on angiogenesis-related genes in this study. This study is the first to our knowledge to develop predictive indicators using a collection of angiogenesis-related genes in patients with LUAD.

Angiogenesis is essential for tissue repair, reproduction, embryonic development, and wound healing, among other physiological functions (Cui et al., 2014). In the normal resting state, endothelial cells can sense angiogenic signals and remain highly plastic under controlled conditions to participate in angiogenesis. However, in many disease states, such as cancer, rheumatoid arthritis, and atherosclerosis, uncontrolled angiogenesis will further promote disease progression and become a hallmark of these disease states (Carmeliet and Jain, 2011). Metastasis and growth of LUAD are similarly dependent on the process of angiogenesis. In hypoxic and energy-deficient conditions, tumor cells and their surroundings produce significant quantities of angiogenic factors, promoting pathological angiogenesis. These new blood vessels will offer oxygen and energy to the tumor and carry metabolic waste generated by the tumor cells, boosting tumor development.

In this study, we collected transcript expression data and corresponding clinical data from TCGA and GEO databases. We determined the differential expression of ARGs in tumor and normal tissues using the TCGA database. GO annotation and KEGG enrichment analysis revealed that these differential genes are primarily engaged in angiogenesis and vascular development and are enriched in the P13K-Akt, Rap1, and MAPK pathways. PI3K/AKT is an essential intracellular signal transduction molecule involved in the control of cell proliferation, apoptosis, and differentiation, and it can affect the production of VEGF and hypoxia-inducible factor (HIF-1) through activation of p70S6K1 and Hdm2 (Skinner et al., 2004). A vascular endothelial growth factor is an essential regulator in tumor angiogenesis, and hypoxia increases the release of angiogenic factors. Furthermore, the PI3K-Akt pathway is critical in K-ras signaling pathway-mediated hematopoiesis and angiogenesis (Liu et al., 2008). The MAPK signaling pathway is one of the essential signaling systems in living creatures, and it is crucial for cell survival, proliferation, and angiogenesis (Teng et al., 2018). Numerous studies have demonstrated that RAP1 is activated in numerous malignancies, including leukemia and solid tumors (Minato, 2013; Shah et al., 2019). aRAP1 plays a role in the invasion and metastasis of various tumor cells by regulating adhesive junctions and cytoskeletal remodeling. DO analysis has shown that these differential genes are associated with lung adenocarcinoma, non-small cell lung cancer, and lung disease.

Subsequently, we used 6 ARGs (MET, CCND2, PDGFB, POSTN, PECAM1, TIMP1) to establish model equations for risk assessment. TIMP1 is a natural inhibitor of matrix metalloproteinases, and the imbalance between TIMP1 and matrix metalloproteinases in gastrointestinal cancers is a crucial element in colon cancer (Song et al., 2016). The interaction of TIMP1 protein’s C-terminal structural domain with tetraspanin CD63 stimulates conformational activation of integrin b1 and activates MAPK signaling, resulting in cancer (Jung et al., 2006). Through the TIMP-1/CD63 signal, TIMP1 can activate fibroblast-like hepatic stellate cells (HSCs) and release SDF-1 to attract neutrophils that promote metastasis. In this process, TIMP1 significantly increases the sensitivity of the liver to circulating tumor cells and creates a tumor microenvironment that promotes tumor liver metastasis (Seubert et al., 2015; Grünwald et al., 2016). A transmembrane receptor tyrosine kinase, MET is the hepatocyte growth factor receptor. When it binds to ligands, it activates several downstream channels that govern cell growth, survival, and migration, among other things (Trusolino et al., 2010). It has been demonstrated that MET interacts with vascular endothelial growth factors to promote neovascularization (Eder et al., 2009). POSTN promotes angiogenesis in colorectal malignancies by preventing stress-induced apoptosis and increasing endothelial cell survival, thereby promoting colon cancer metastasis, leading to poor prognosis (Bao et al., 2004). In some tumors, such as glioma (Hermanson et al., 1992; Guo et al., 2003) and ovarian cancer (Czekierdowska et al., 2017), PDGFB plays a crucial role in promoting angiogenesis and stimulating malignant cell proliferation. PECAM-1 (also referred to as a cluster of differentiation 31, CD31) is primarily regarded as an adhesion molecule that participates in cell proliferation, apoptosis, migration, and cellular immunity. PECAM-1 is expressed in some tumor cells and has been linked to tumor invasion (Ranamukhaarachchi et al., 2019; Gong et al., 2020). CCND2 encodes cell cycle protein D2, which regulates cell cycle protein-dependent kinases 4 and 6 (CDK) 4/6 in G1-S (Ortega et al., 2002). Although CCND2 dysregulation is a significant source of medication resistance in breast cancer endocrine treatment (Kwapisz, 2017), its significance in LUAD remains unknown. The role of these genes in LUAD needs to be further investigated. Subsequently, through a series of validations and exploration of possible related mechanisms, we demonstrated that the risk model we constructed could predict the prognosis of LUAD patients.

Our study certainly has some limitations. To some extent, intra-tumor or intra-patient tumor heterogeneity is unavoidable, given that our study cohort was compiled from a wide variety of high-throughput sequencing platforms and public databases. Several studies demonstrate that the heterogeneity of tumors impacts the effectiveness of immunotherapy and chemotherapy. We must disregard the considerable heterogeneity of lung adenocarcinoma due to data constraints. Second, although it has been discovered that angiogenesis-related genes influence immunological interactions and survival in LUAD patients, the scientific or medical processes behind these occurrences are not fully understood. To validate and explain the significance of angiogenesis-related genes in LUAD, therefore, large-scale prospective research and functional and mechanistic tests are required. Third, while the median RS cut-off point was utilized to categorize LUAD samples into HR and HR groups, the ideal RS cut-off point may be a preferred technique for stratifying LUAD patients. Due to the lack of comprehensive clinicopathological data, we finally compiled and adjusted certain clinical data for survival analysis and Cox regression. However, this may introduce bias and uncertainty regarding whether RS is an independent predictive factor.

Non-etheless, our research identifies genes and pathways implicated in LUAD angiogenesis and develops a valid predictive model that correlates with immune infiltration features and treatment. The RS model contributes to a greater understanding of the prognosis of LUAD patients and opens up new perspectives for targeted therapy.

## Data Availability

The original contributions presented in the study are included in the article/Supplementary Material, further inquiries can be directed to the corresponding authors.
