# Supplementary figures and images for: Application of an angiogenesis-related genes risk model in lung adenocarcinoma prognosis and immunotherapy
Source: Front Genet. 2023 Feb 1;14:1092968. doi: 10.3389/fgene.2023.1092968 (PMC9929558; doi:10.3389/fgene.2023.1092968)

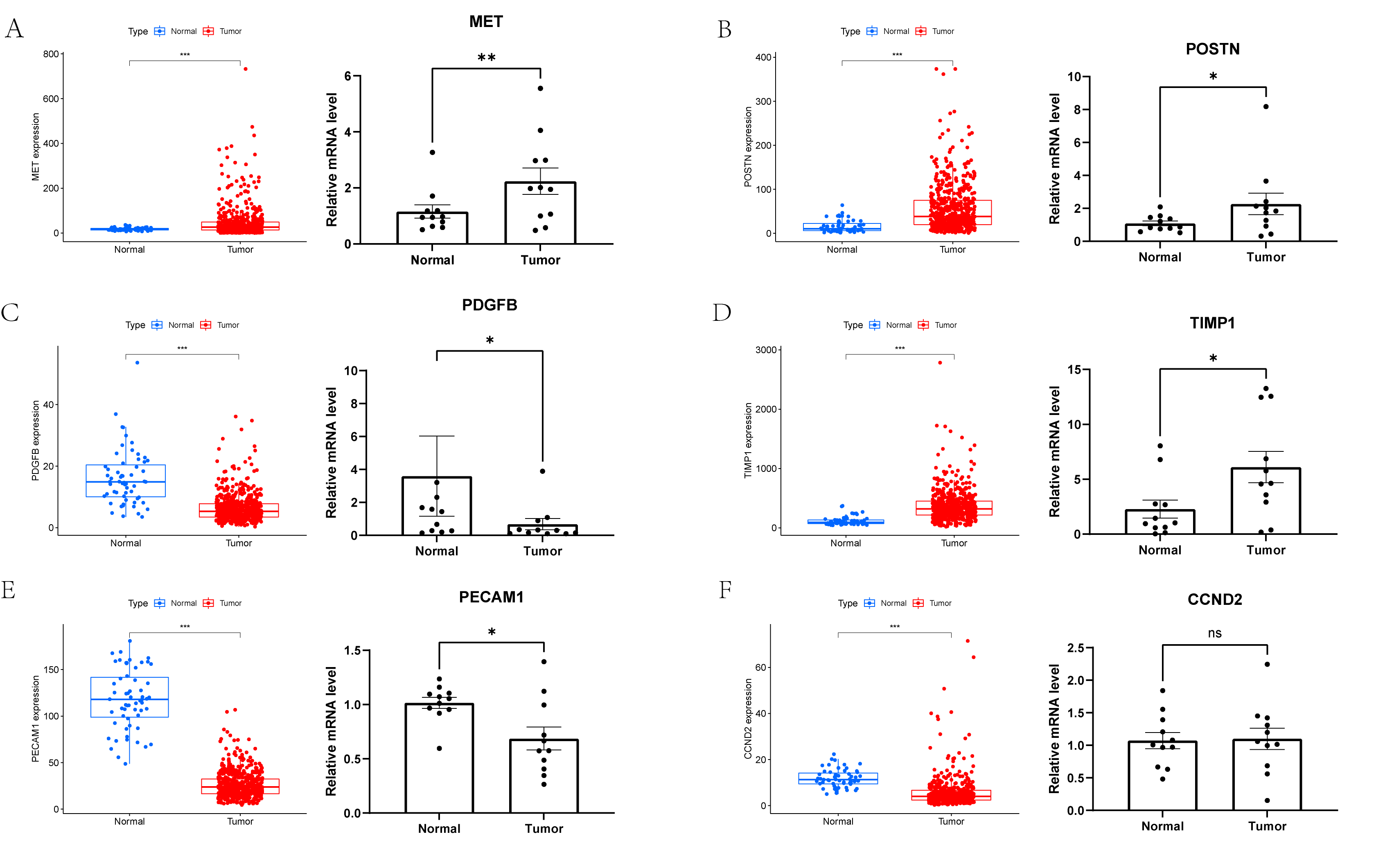

Supplement: Supplementary file 1 [file Image6.tif]

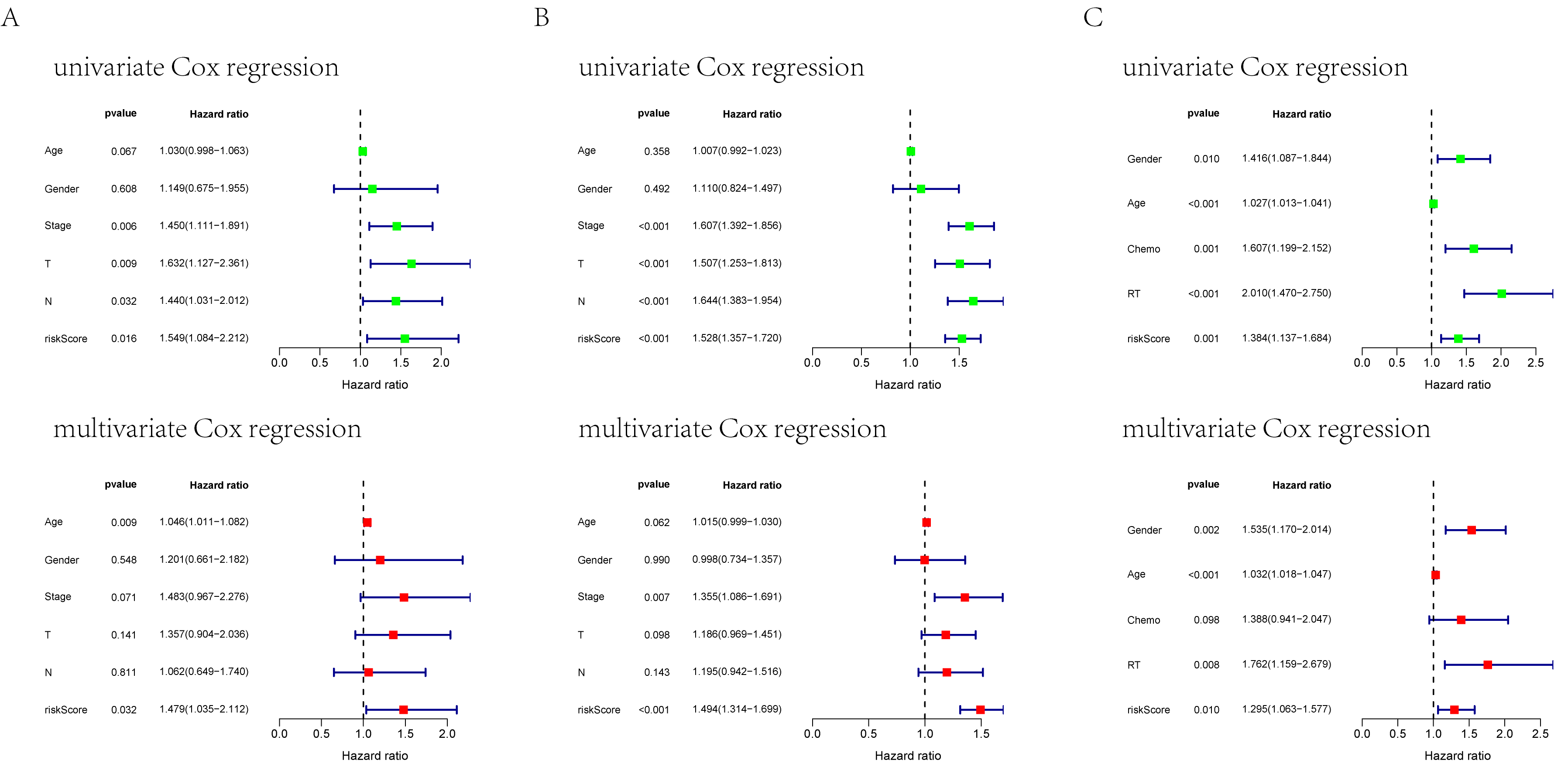

Supplement: Supplementary file 3 [file Image3.tif]

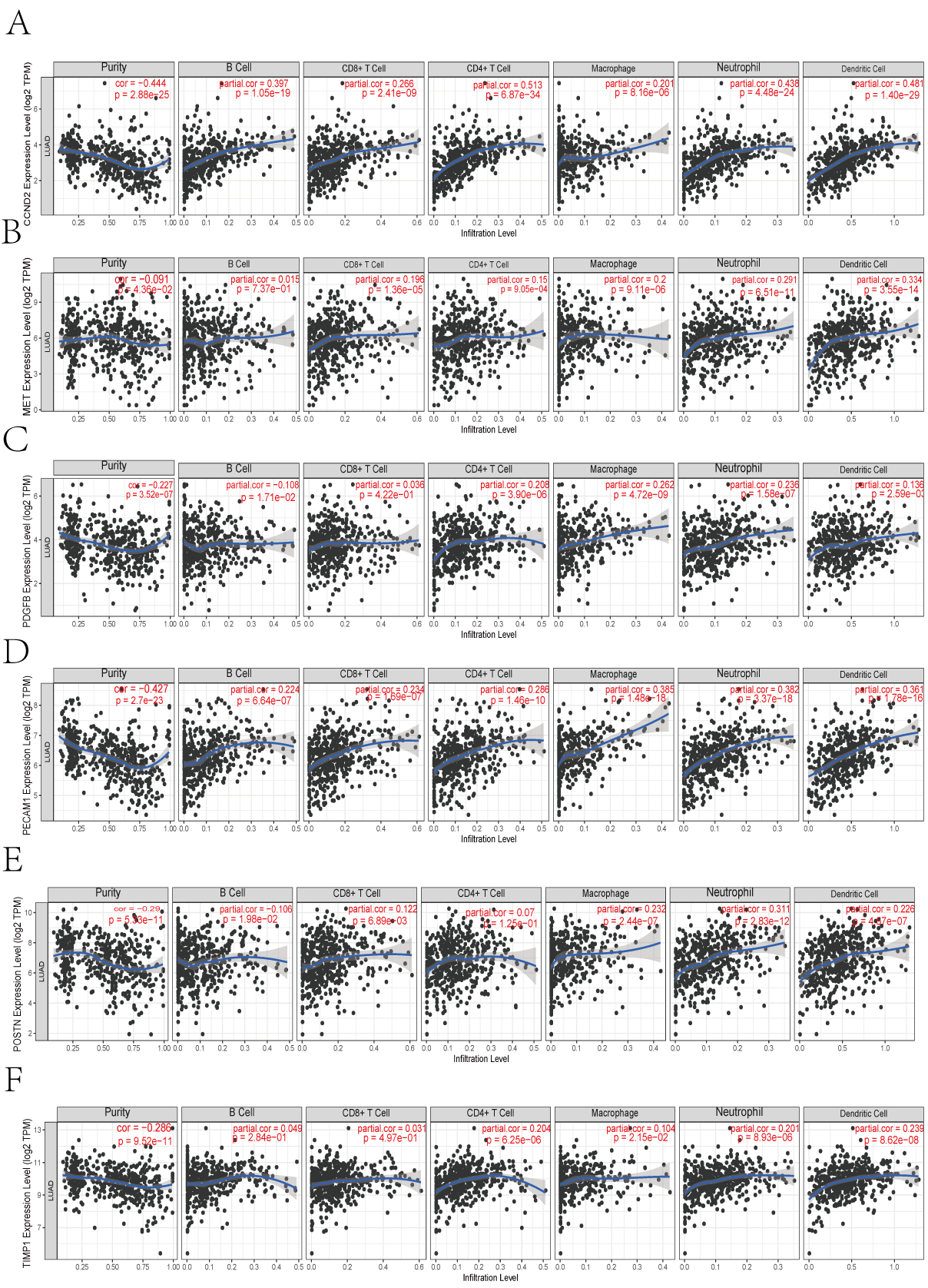

Supplement: Supplementary file 4 [file Image4.TIF]

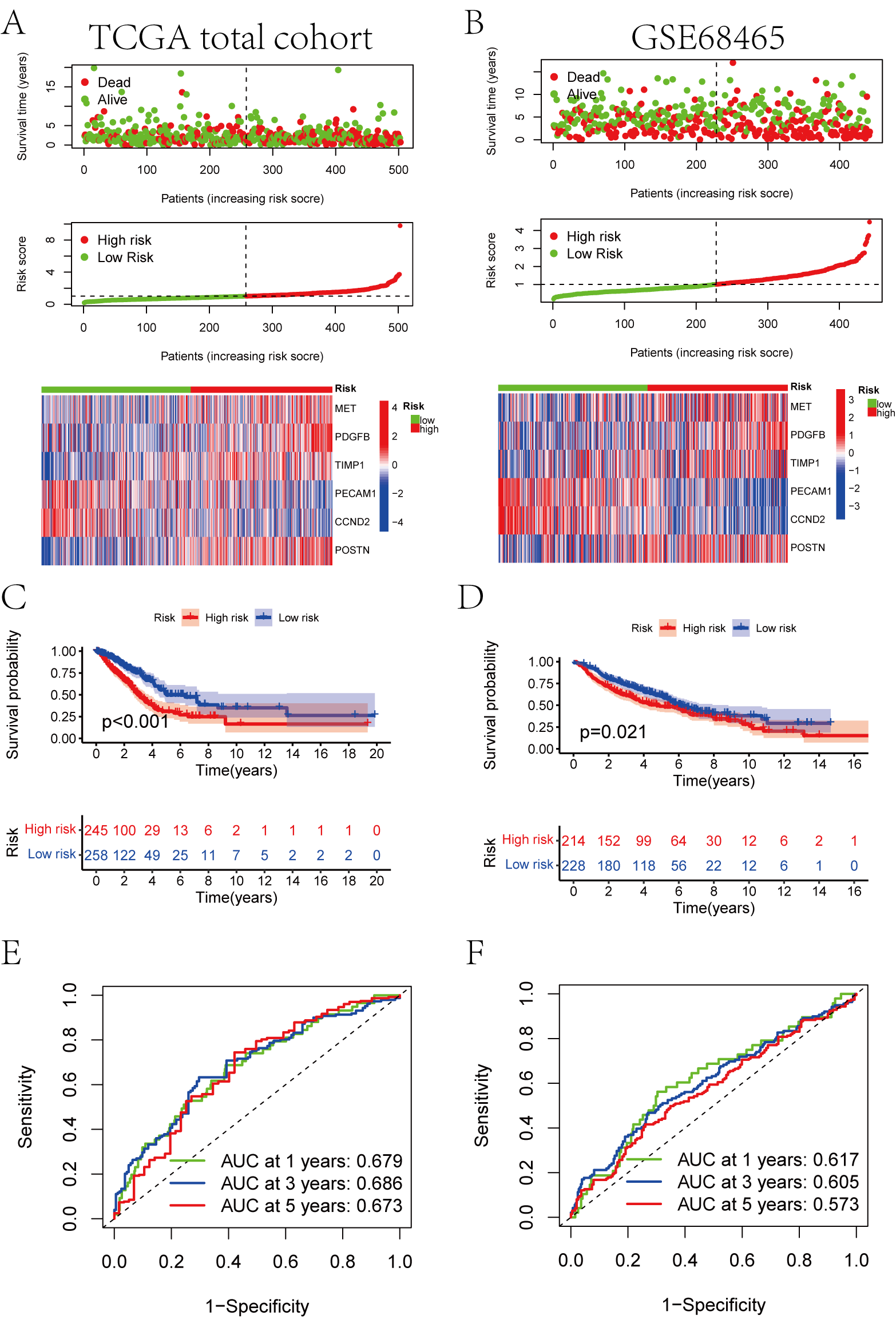

Supplement: Supplementary file 5 [file Image2.tif]

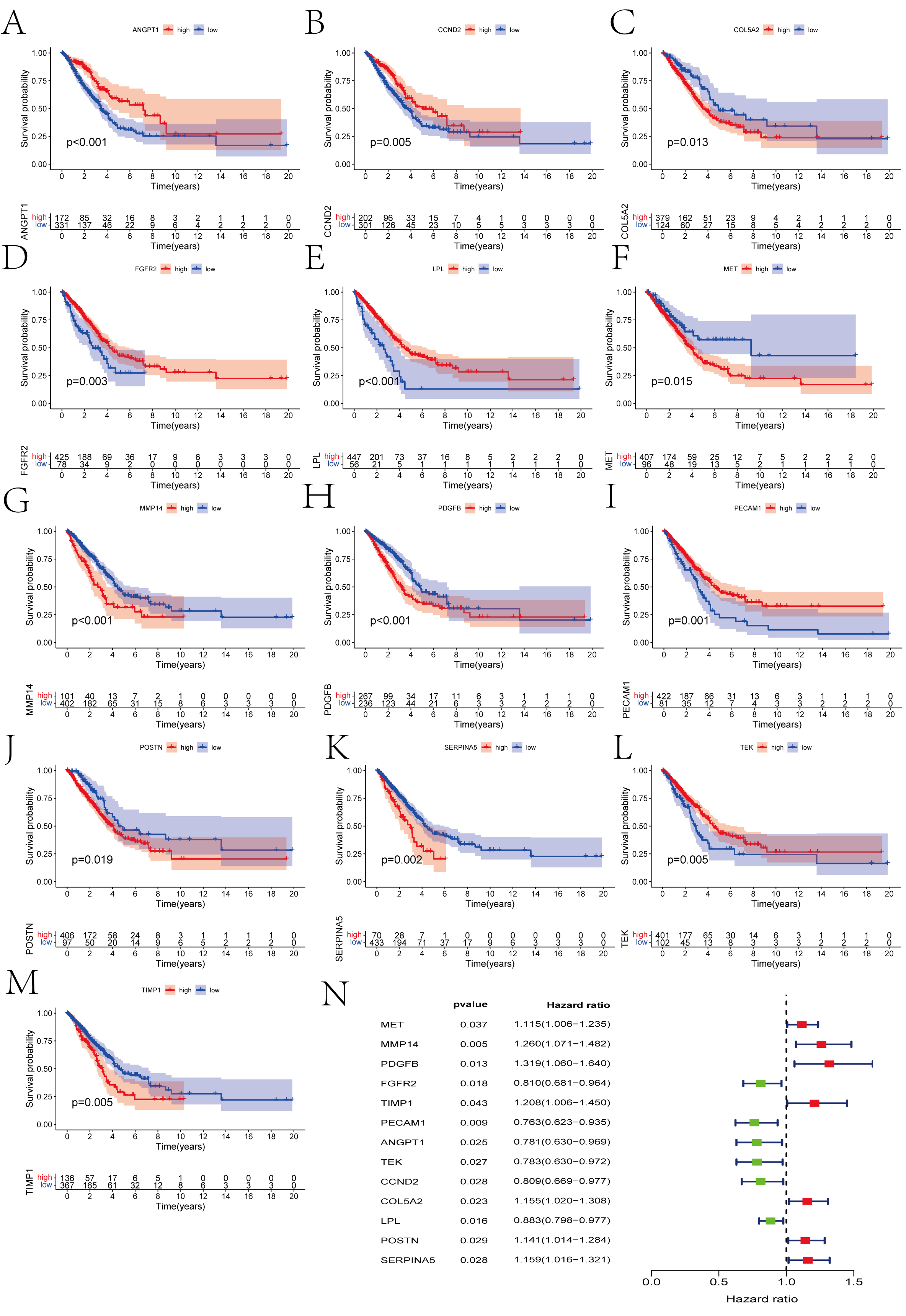

Supplement: Supplementary file 6 [file Image1.TIF]

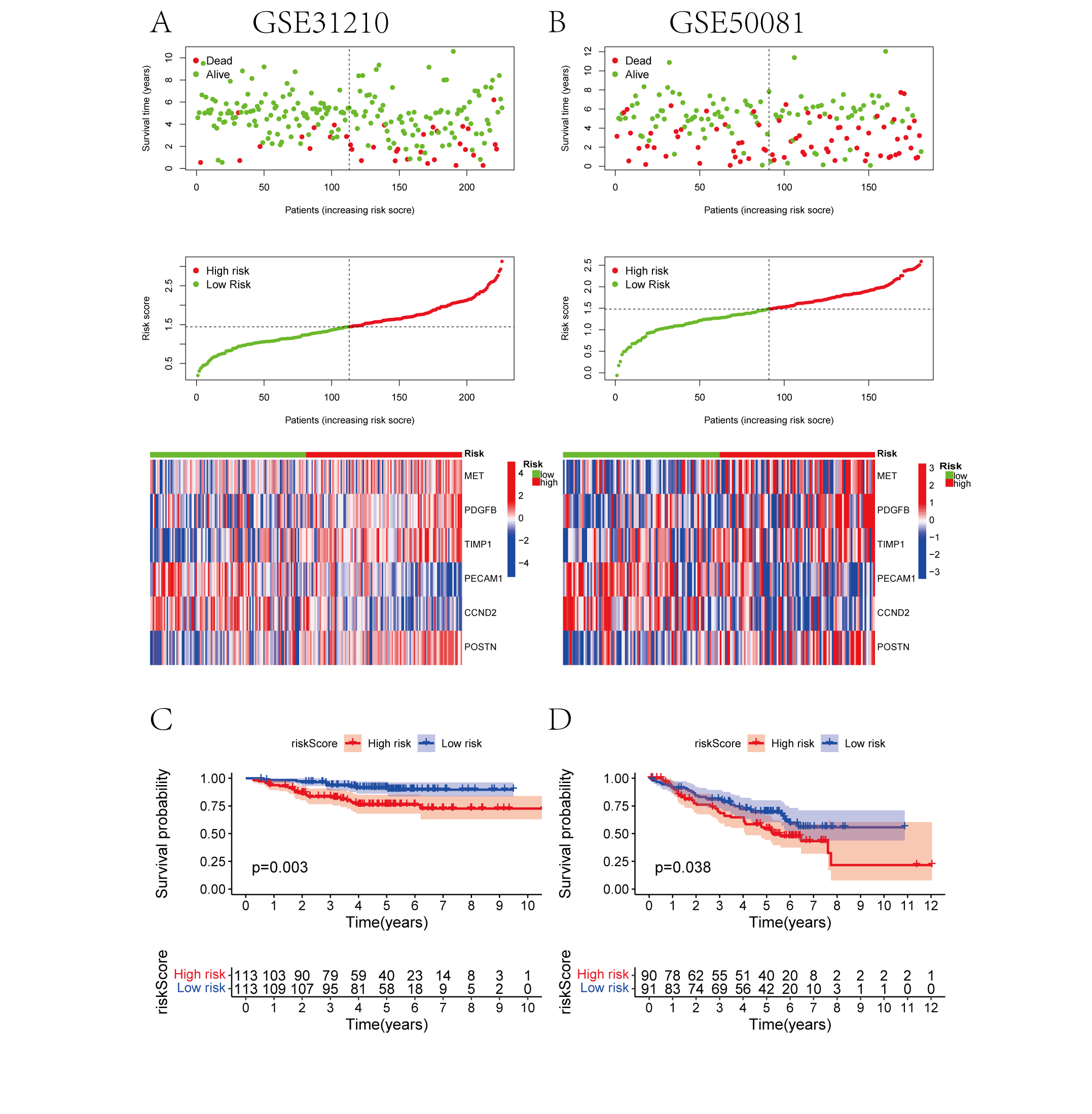

Supplement: Supplementary file 10 [file Image5.tif]
