# Supplementary material for: Application of an angiogenesis-related genes risk model in lung adenocarcinoma prognosis and immunotherapy
Source: Front Genet. 2023 Feb 1;14:1092968. doi: 10.3389/fgene.2023.1092968 (PMC9929558; doi:10.3389/fgene.2023.1092968)
Supplement: Supplementary file 2 [file Table1.DOCX]

Table S1 The information of DO enrichment analysis

| ID | Description | GeneRatio | BgRatio | pvalue | p.adjust | qvalue |
| --- | --- | --- | --- | --- | --- | --- |
| DOID:854 | collagen disease | 17/54 | 201/8007 | 6.61E-15 | 2.07E-12 | 8.77E-13 |
| DOID:5844 | myocardial infarction | 19/54 | 279/8007 | 6.67E-15 | 2.07E-12 | 8.77E-13 |
| DOID:13207 | proliferative diabetic retinopathy | 10/54 | 35/8007 | 1.31E-14 | 2.46E-12 | 1.04E-12 |
| DOID:3996 | urinary system cancer | 23/54 | 500/8007 | 2.13E-14 | 2.46E-12 | 1.04E-12 |
| DOID:1936 | atherosclerosis | 20/54 | 344/8007 | 2.25E-14 | 2.46E-12 | 1.04E-12 |
| DOID:2348 | arteriosclerotic cardiovascular disease | 20/54 | 345/8007 | 2.38E-14 | 2.46E-12 | 1.04E-12 |
| DOID:3393 | coronary artery disease | 20/54 | 348/8007 | 2.81E-14 | 2.49E-12 | 1.06E-12 |
| DOID:2349 | arteriosclerosis | 20/54 | 356/8007 | 4.33E-14 | 3.36E-12 | 1.43E-12 |
| DOID:4451 | renal carcinoma | 20/54 | 380/8007 | 1.50E-13 | 1.03E-11 | 4.37E-12 |
| DOID:263 | kidney cancer | 21/54 | 437/8007 | 1.83E-13 | 1.13E-11 | 4.81E-12 |
| DOID:850 | lung disease | 22/54 | 499/8007 | 2.32E-13 | 1.31E-11 | 5.55E-12 |
| DOID:1575 | rheumatic disease | 15/54 | 188/8007 | 8.07E-13 | 3.57E-11 | 1.52E-11 |
| DOID:418 | systemic scleroderma | 15/54 | 188/8007 | 8.07E-13 | 3.57E-11 | 1.52E-11 |
| DOID:419 | scleroderma | 15/54 | 188/8007 | 8.07E-13 | 3.57E-11 | 1.52E-11 |
| DOID:0060084 | cell type benign neoplasm | 21/54 | 475/8007 | 9.35E-13 | 3.87E-11 | 1.64E-11 |
| DOID:2462 | retinal vascular disease | 10/54 | 58/8007 | 3.31E-12 | 1.18E-10 | 5.01E-11 |
| DOID:8947 | diabetic retinopathy | 10/54 | 58/8007 | 3.31E-12 | 1.18E-10 | 5.01E-11 |
| DOID:4450 | renal cell carcinoma | 18/54 | 342/8007 | 3.42E-12 | 1.18E-10 | 5.01E-11 |
| DOID:6432 | pulmonary hypertension | 10/54 | 77/8007 | 6.32E-11 | 2.06E-09 | 8.75E-10 |
| DOID:201 | connective tissue cancer | 17/54 | 370/8007 | 1.36E-10 | 4.23E-09 | 1.79E-09 |
| DOID:6713 | cerebrovascular disease | 11/54 | 116/8007 | 2.07E-10 | 6.11E-09 | 2.60E-09 |
| DOID:10591 | pre-eclampsia | 15/54 | 278/8007 | 2.26E-10 | 6.37E-09 | 2.70E-09 |
| DOID:9408 | acute myocardial infarction | 10/54 | 92/8007 | 3.85E-10 | 1.04E-08 | 4.40E-09 |
| DOID:184 | bone cancer | 14/54 | 243/8007 | 4.26E-10 | 1.10E-08 | 4.67E-09 |
| DOID:3963 | thyroid carcinoma | 13/54 | 200/8007 | 4.50E-10 | 1.12E-08 | 4.74E-09 |
| DOID:1781 | thyroid cancer | 13/54 | 213/8007 | 9.82E-10 | 2.34E-08 | 9.94E-09 |
| DOID:12365 | malaria | 10/54 | 103/8007 | 1.19E-09 | 2.74E-08 | 1.16E-08 |
| DOID:0070004 | myeloma | 15/54 | 323/8007 | 1.83E-09 | 4.06E-08 | 1.72E-08 |
| DOID:0060100 | musculoskeletal system cancer | 17/54 | 439/8007 | 1.93E-09 | 4.13E-08 | 1.75E-08 |
| DOID:3459 | breast carcinoma | 16/54 | 383/8007 | 2.19E-09 | 4.52E-08 | 1.92E-08 |
| DOID:4960 | bone marrow cancer | 15/54 | 328/8007 | 2.26E-09 | 4.53E-08 | 1.92E-08 |
| DOID:120 | female reproductive organ cancer | 17/54 | 455/8007 | 3.34E-09 | 6.47E-08 | 2.74E-08 |
| DOID:557 | kidney disease | 17/54 | 461/8007 | 4.07E-09 | 7.65E-08 | 3.25E-08 |
| DOID:18 | urinary system disease | 17/54 | 478/8007 | 7.05E-09 | 1.29E-07 | 5.45E-08 |
| DOID:2789 | parasitic protozoa infectious disease | 10/54 | 127/8007 | 9.35E-09 | 1.66E-07 | 7.03E-08 |
| DOID:127 | leiomyoma | 9/54 | 95/8007 | 1.09E-08 | 1.87E-07 | 7.94E-08 |
| DOID:3908 | non-small cell lung carcinoma | 16/54 | 431/8007 | 1.20E-08 | 2.01E-07 | 8.52E-08 |
| DOID:3347 | osteosarcoma | 12/54 | 215/8007 | 1.31E-08 | 2.14E-07 | 9.10E-08 |
| DOID:10534 | stomach cancer | 13/54 | 274/8007 | 2.10E-08 | 3.33E-07 | 1.41E-07 |
| DOID:3969 | papillary thyroid carcinoma | 9/54 | 108/8007 | 3.39E-08 | 4.92E-07 | 2.09E-07 |
| DOID:552 | pneumonia | 9/54 | 108/8007 | 3.39E-08 | 4.92E-07 | 2.09E-07 |
| DOID:2151 | malignant ovarian surface epithelial-stromal neoplasm | 13/54 | 286/8007 | 3.49E-08 | 4.92E-07 | 2.09E-07 |
| DOID:2152 | ovary epithelial cancer | 13/54 | 286/8007 | 3.49E-08 | 4.92E-07 | 2.09E-07 |
| DOID:4001 | ovarian carcinoma | 13/54 | 286/8007 | 3.49E-08 | 4.92E-07 | 2.09E-07 |
| DOID:1115 | sarcoma | 11/54 | 193/8007 | 4.65E-08 | 6.28E-07 | 2.66E-07 |
| DOID:1398 | parasitic infectious disease | 10/54 | 150/8007 | 4.66E-08 | 6.28E-07 | 2.66E-07 |
| DOID:2213 | hemorrhagic disease | 10/54 | 152/8007 | 5.28E-08 | 6.97E-07 | 2.96E-07 |
| DOID:5679 | retinal disease | 14/54 | 358/8007 | 6.30E-08 | 8.13E-07 | 3.45E-07 |
| DOID:4989 | pancreatitis | 9/54 | 117/8007 | 6.83E-08 | 8.64E-07 | 3.67E-07 |
| DOID:10283 | prostate cancer | 15/54 | 425/8007 | 7.50E-08 | 9.30E-07 | 3.95E-07 |
| DOID:326 | ischemia | 11/54 | 204/8007 | 8.23E-08 | 1.00E-06 | 4.25E-07 |
| DOID:13241 | Behcet's disease | 8/54 | 86/8007 | 8.86E-08 | 1.06E-06 | 4.48E-07 |
| DOID:3070 | malignant glioma | 11/54 | 207/8007 | 9.55E-08 | 1.11E-06 | 4.70E-07 |
| DOID:2394 | ovarian cancer | 13/54 | 312/8007 | 9.76E-08 | 1.11E-06 | 4.70E-07 |
| DOID:865 | vasculitis | 9/54 | 122/8007 | 9.83E-08 | 1.11E-06 | 4.70E-07 |
| DOID:3856 | male reproductive organ cancer | 15/54 | 435/8007 | 1.02E-07 | 1.13E-06 | 4.79E-07 |
| DOID:3770 | pulmonary fibrosis | 9/54 | 123/8007 | 1.06E-07 | 1.15E-06 | 4.87E-07 |
| DOID:3082 | interstitial lung disease | 10/54 | 170/8007 | 1.53E-07 | 1.63E-06 | 6.92E-07 |
| DOID:3083 | chronic obstructive pulmonary disease | 11/54 | 220/8007 | 1.78E-07 | 1.87E-06 | 7.93E-07 |
| DOID:1036 | chronic leukemia | 11/54 | 223/8007 | 2.04E-07 | 2.11E-06 | 8.95E-07 |
| DOID:4607 | biliary tract cancer | 10/54 | 178/8007 | 2.35E-07 | 2.39E-06 | 1.01E-06 |
| DOID:0060122 | integumentary system cancer | 8/54 | 98/8007 | 2.47E-07 | 2.43E-06 | 1.03E-06 |
| DOID:4159 | skin cancer | 8/54 | 98/8007 | 2.47E-07 | 2.43E-06 | 1.03E-06 |
| DOID:1247 | blood coagulation disease | 10/54 | 180/8007 | 2.61E-07 | 2.53E-06 | 1.07E-06 |
| DOID:10952 | nephritis | 9/54 | 147/8007 | 4.88E-07 | 4.65E-06 | 1.98E-06 |
| DOID:2320 | obstructive lung disease | 12/54 | 308/8007 | 6.80E-07 | 6.39E-06 | 2.71E-06 |
| DOID:4971 | myelofibrosis | 6/54 | 49/8007 | 7.92E-07 | 7.22E-06 | 3.07E-06 |
| DOID:7693 | abdominal aortic aneurysm | 6/54 | 49/8007 | 7.92E-07 | 7.22E-06 | 3.07E-06 |
| DOID:2921 | glomerulonephritis | 7/54 | 79/8007 | 8.48E-07 | 7.62E-06 | 3.23E-06 |
| DOID:10286 | prostate carcinoma | 8/54 | 122/8007 | 1.34E-06 | 1.18E-05 | 5.02E-06 |
| DOID:5082 | liver cirrhosis | 10/54 | 220/8007 | 1.66E-06 | 1.45E-05 | 6.15E-06 |
| DOID:1040 | chronic lymphocytic leukemia | 10/54 | 221/8007 | 1.73E-06 | 1.47E-05 | 6.26E-06 |
| DOID:26 | pancreas disease | 9/54 | 171/8007 | 1.74E-06 | 1.47E-05 | 6.26E-06 |
| DOID:13001 | carotid stenosis | 4/54 | 14/8007 | 1.76E-06 | 1.47E-05 | 6.26E-06 |
| DOID:4896 | bile duct adenocarcinoma | 8/54 | 128/8007 | 1.92E-06 | 1.57E-05 | 6.66E-06 |
| DOID:4947 | cholangiocarcinoma | 8/54 | 128/8007 | 1.92E-06 | 1.57E-05 | 6.66E-06 |
| DOID:3146 | lipid metabolism disorder | 7/54 | 92/8007 | 2.40E-06 | 1.93E-05 | 8.19E-06 |
| DOID:3910 | lung adenocarcinoma | 9/54 | 178/8007 | 2.44E-06 | 1.94E-05 | 8.22E-06 |
| DOID:3627 | aortic aneurysm | 6/54 | 60/8007 | 2.68E-06 | 2.10E-05 | 8.93E-06 |
| DOID:520 | aortic disease | 6/54 | 61/8007 | 2.96E-06 | 2.29E-05 | 9.72E-06 |
| DOID:1542 | head and neck carcinoma | 10/54 | 235/8007 | 3.01E-06 | 2.31E-05 | 9.79E-06 |
| DOID:2218 | blood platelet disease | 7/54 | 96/8007 | 3.19E-06 | 2.40E-05 | 1.02E-05 |
| DOID:12716 | newborn respiratory distress syndrome | 5/54 | 35/8007 | 3.22E-06 | 2.40E-05 | 1.02E-05 |
| DOID:3454 | brain infarction | 6/54 | 62/8007 | 3.26E-06 | 2.40E-05 | 1.02E-05 |
| DOID:11934 | head and neck cancer | 10/54 | 239/8007 | 3.51E-06 | 2.56E-05 | 1.09E-05 |
| DOID:3388 | periodontal disease | 8/54 | 139/8007 | 3.58E-06 | 2.58E-05 | 1.09E-05 |
| DOID:4606 | bile duct cancer | 8/54 | 140/8007 | 3.77E-06 | 2.66E-05 | 1.13E-05 |
| DOID:4897 | bile duct carcinoma | 8/54 | 140/8007 | 3.77E-06 | 2.66E-05 | 1.13E-05 |
| DOID:2237 | hepatitis | 13/54 | 431/8007 | 3.88E-06 | 2.71E-05 | 1.15E-05 |
| DOID:75 | lymphatic system disease | 7/54 | 101/8007 | 4.49E-06 | 3.09E-05 | 1.31E-05 |
| DOID:1037 | lymphoblastic leukemia | 13/54 | 443/8007 | 5.26E-06 | 3.58E-05 | 1.52E-05 |
| DOID:0050700 | cardiomyopathy | 8/54 | 149/8007 | 6.01E-06 | 4.05E-05 | 1.72E-05 |
| DOID:3407 | carotid artery disease | 4/54 | 19/8007 | 6.64E-06 | 4.40E-05 | 1.87E-05 |
| DOID:3068 | glioblastoma multiforme | 6/54 | 70/8007 | 6.66E-06 | 4.40E-05 | 1.87E-05 |
| DOID:5517 | stomach carcinoma | 8/54 | 153/8007 | 7.31E-06 | 4.77E-05 | 2.03E-05 |
| DOID:9538 | multiple myeloma | 10/54 | 264/8007 | 8.51E-06 | 5.49E-05 | 2.33E-05 |
| DOID:3069 | astrocytoma | 7/54 | 112/8007 | 8.94E-06 | 5.71E-05 | 2.42E-05 |
| DOID:0060095 | uterine benign neoplasm | 5/54 | 43/8007 | 9.15E-06 | 5.73E-05 | 2.43E-05 |
| DOID:13223 | uterine fibroid | 5/54 | 43/8007 | 9.15E-06 | 5.73E-05 | 2.43E-05 |
| DOID:28 | endocrine system disease | 12/54 | 399/8007 | 1.01E-05 | 6.28E-05 | 2.67E-05 |
| DOID:74 | hematopoietic system disease | 13/54 | 474/8007 | 1.10E-05 | 6.74E-05 | 2.86E-05 |
| DOID:1091 | tooth disease | 8/54 | 162/8007 | 1.11E-05 | 6.77E-05 | 2.87E-05 |
| DOID:0060086 | female reproductive organ benign neoplasm | 5/54 | 45/8007 | 1.15E-05 | 6.92E-05 | 2.94E-05 |
| DOID:0050622 | reproductive organ benign neoplasm | 5/54 | 47/8007 | 1.43E-05 | 8.52E-05 | 3.62E-05 |
| DOID:1168 | familial hyperlipidemia | 6/54 | 82/8007 | 1.67E-05 | 9.79E-05 | 4.15E-05 |
| DOID:2871 | endometrial carcinoma | 6/54 | 82/8007 | 1.67E-05 | 9.79E-05 | 4.15E-05 |
| DOID:3620 | central nervous system cancer | 7/54 | 124/8007 | 1.75E-05 | 0.000101 | 4.29E-05 |
| DOID:5520 | head and neck squamous cell carcinoma | 8/54 | 174/8007 | 1.88E-05 | 0.000108 | 4.58E-05 |
| DOID:14069 | cerebral malaria | 4/54 | 25/8007 | 2.10E-05 | 0.00012 | 5.08E-05 |
| DOID:4079 | heart valve disease | 5/54 | 51/8007 | 2.14E-05 | 0.000121 | 5.13E-05 |
| DOID:3978 | extrinsic cardiomyopathy | 4/54 | 26/8007 | 2.47E-05 | 0.000137 | 5.80E-05 |
| DOID:820 | myocarditis | 4/54 | 26/8007 | 2.47E-05 | 0.000137 | 5.80E-05 |
| DOID:289 | endometriosis | 6/54 | 88/8007 | 2.51E-05 | 0.000137 | 5.80E-05 |
| DOID:2916 | hypersensitivity reaction type IV disease | 6/54 | 88/8007 | 2.51E-05 | 0.000137 | 5.80E-05 |
| DOID:0060036 | intrinsic cardiomyopathy | 7/54 | 135/8007 | 3.03E-05 | 0.000163 | 6.94E-05 |
| DOID:11162 | respiratory failure | 5/54 | 55/8007 | 3.11E-05 | 0.000166 | 7.06E-05 |
| DOID:403 | mouth disease | 8/54 | 188/8007 | 3.28E-05 | 0.000174 | 7.39E-05 |
| DOID:3962 | follicular thyroid carcinoma | 5/54 | 56/8007 | 3.40E-05 | 0.000179 | 7.58E-05 |
| DOID:2645 | benign mesothelioma | 5/54 | 57/8007 | 3.71E-05 | 0.000193 | 8.20E-05 |
| DOID:229 | female reproductive system disease | 8/54 | 193/8007 | 3.96E-05 | 0.000205 | 8.69E-05 |
| DOID:3451 | skin carcinoma | 5/54 | 58/8007 | 4.04E-05 | 0.000207 | 8.78E-05 |
| DOID:1380 | endometrial cancer | 6/54 | 97/8007 | 4.37E-05 | 0.000222 | 9.43E-05 |
| DOID:11400 | pyelonephritis | 3/54 | 11/8007 | 4.61E-05 | 0.000226 | 9.60E-05 |
| DOID:2744 | pyelitis | 3/54 | 11/8007 | 4.61E-05 | 0.000226 | 9.60E-05 |
| DOID:4465 | papillary renal cell carcinoma | 3/54 | 11/8007 | 4.61E-05 | 0.000226 | 9.60E-05 |
| DOID:3498 | pancreatic ductal adenocarcinoma | 6/54 | 98/8007 | 4.63E-05 | 0.000226 | 9.60E-05 |
| DOID:363 | uterine cancer | 6/54 | 98/8007 | 4.63E-05 | 0.000226 | 9.60E-05 |
| DOID:16 | integumentary system disease | 11/54 | 394/8007 | 5.02E-05 | 0.000242 | 0.000103 |
| DOID:5100 | middle ear disease | 4/54 | 31/8007 | 5.08E-05 | 0.000242 | 0.000103 |
| DOID:9744 | type 1 diabetes mellitus | 4/54 | 31/8007 | 5.08E-05 | 0.000242 | 0.000103 |
| DOID:1192 | peripheral nervous system neoplasm | 11/54 | 396/8007 | 5.26E-05 | 0.000249 | 0.000106 |
| DOID:0060085 | organ system benign neoplasm | 9/54 | 262/8007 | 5.48E-05 | 0.000255 | 0.000108 |
| DOID:423 | myopathy | 11/54 | 398/8007 | 5.51E-05 | 0.000255 | 0.000108 |
| DOID:66 | muscle tissue disease | 11/54 | 398/8007 | 5.51E-05 | 0.000255 | 0.000108 |
| DOID:655 | inherited metabolic disorder | 10/54 | 331/8007 | 6.01E-05 | 0.000276 | 0.000117 |
| DOID:3355 | fibrosarcoma | 4/54 | 33/8007 | 6.54E-05 | 0.000298 | 0.000127 |
| DOID:0080000 | muscular disease | 11/54 | 408/8007 | 6.90E-05 | 0.000312 | 0.000133 |
| DOID:104 | bacterial infectious disease | 9/54 | 271/8007 | 7.13E-05 | 0.00032 | 0.000136 |
| DOID:2994 | germ cell cancer | 11/54 | 410/8007 | 7.21E-05 | 0.000322 | 0.000137 |
| DOID:332 | amyotrophic lateral sclerosis | 7/54 | 155/8007 | 7.34E-05 | 0.000324 | 0.000138 |
| DOID:10223 | dermatomyositis | 4/54 | 34/8007 | 7.38E-05 | 0.000324 | 0.000138 |
| DOID:11054 | urinary bladder cancer | 6/54 | 107/8007 | 7.59E-05 | 0.000331 | 0.000141 |
| DOID:9352 | type 2 diabetes mellitus | 8/54 | 215/8007 | 8.52E-05 | 0.000369 | 0.000157 |
| DOID:1588 | thrombocytopenia | 5/54 | 68/8007 | 8.72E-05 | 0.000373 | 0.000158 |
| DOID:576 | proteinuria | 5/54 | 68/8007 | 8.72E-05 | 0.000373 | 0.000158 |
| DOID:230 | lateral sclerosis | 6/54 | 111/8007 | 9.32E-05 | 0.000394 | 0.000167 |
| DOID:4074 | pancreas adenocarcinoma | 7/54 | 161/8007 | 9.33E-05 | 0.000394 | 0.000167 |
| DOID:37 | skin disease | 10/54 | 350/8007 | 9.59E-05 | 0.000402 | 0.000171 |
| DOID:1003 | pelvic inflammatory disease | 3/54 | 14/8007 | 0.0001 | 0.000411 | 0.000175 |
| DOID:10140 | dry eye syndrome | 3/54 | 14/8007 | 0.0001 | 0.000411 | 0.000175 |
| DOID:1400 | lacrimal apparatus disease | 3/54 | 14/8007 | 0.0001 | 0.000411 | 0.000175 |
| DOID:3326 | purpura | 5/54 | 71/8007 | 0.000107 | 0.000438 | 0.000186 |
| DOID:11612 | polycystic ovary syndrome | 7/54 | 166/8007 | 0.000113 | 0.000457 | 0.000194 |
| DOID:10747 | lymphoid leukemia | 5/54 | 72/8007 | 0.000115 | 0.000457 | 0.000194 |
| DOID:5409 | lung small cell carcinoma | 5/54 | 72/8007 | 0.000115 | 0.000457 | 0.000194 |
| DOID:783 | end stage renal failure | 4/54 | 38/8007 | 0.000115 | 0.000457 | 0.000194 |
| DOID:6000 | congestive heart failure | 8/54 | 229/8007 | 0.000132 | 0.000523 | 0.000222 |
| DOID:0060119 | pharynx cancer | 4/54 | 40/8007 | 0.000141 | 0.000554 | 0.000235 |
| DOID:824 | periodontitis | 6/54 | 120/8007 | 0.000143 | 0.00056 | 0.000237 |
| DOID:11729 | Lyme disease | 3/54 | 16/8007 | 0.000153 | 0.000588 | 0.000249 |
| DOID:61 | mitral valve disease | 3/54 | 16/8007 | 0.000153 | 0.000588 | 0.000249 |
| DOID:1790 | malignant mesothelioma | 4/54 | 41/8007 | 0.000156 | 0.000592 | 0.000251 |
| DOID:5157 | benign pleural mesothelioma | 4/54 | 41/8007 | 0.000156 | 0.000592 | 0.000251 |
| DOID:688 | embryonal cancer | 10/54 | 373/8007 | 0.000162 | 0.000614 | 0.000261 |
| DOID:2621 | autonomic nervous system neoplasm | 10/54 | 375/8007 | 0.00017 | 0.000634 | 0.000269 |
| DOID:769 | neuroblastoma | 10/54 | 375/8007 | 0.00017 | 0.000634 | 0.000269 |
| DOID:1319 | brain cancer | 4/54 | 42/8007 | 0.000171 | 0.000635 | 0.00027 |
| DOID:0050338 | primary bacterial infectious disease | 8/54 | 238/8007 | 0.000173 | 0.000638 | 0.000271 |
| DOID:10941 | intracranial aneurysm | 3/54 | 17/8007 | 0.000184 | 0.000669 | 0.000284 |
| DOID:10976 | membranous glomerulonephritis | 3/54 | 17/8007 | 0.000184 | 0.000669 | 0.000284 |
| DOID:9675 | pulmonary emphysema | 3/54 | 17/8007 | 0.000184 | 0.000669 | 0.000284 |
| DOID:633 | myositis | 5/54 | 81/8007 | 0.000201 | 0.000723 | 0.000307 |
| DOID:15 | reproductive system disease | 10/54 | 386/8007 | 0.000215 | 0.00077 | 0.000327 |
| DOID:4248 | coronary stenosis | 3/54 | 18/8007 | 0.00022 | 0.000785 | 0.000333 |
| DOID:231 | motor neuron disease | 7/54 | 187/8007 | 0.000237 | 0.000834 | 0.000354 |
| DOID:8398 | osteoarthritis | 7/54 | 187/8007 | 0.000237 | 0.000834 | 0.000354 |
| DOID:11335 | sarcoidosis | 5/54 | 84/8007 | 0.000238 | 0.000834 | 0.000354 |
| DOID:2986 | IgA glomerulonephritis | 3/54 | 19/8007 | 0.00026 | 0.000907 | 0.000385 |
| DOID:345 | uterine disease | 4/54 | 48/8007 | 0.000289 | 0.001 | 0.000424 |
| DOID:3302 | chordoma | 3/54 | 20/8007 | 0.000305 | 0.001044 | 0.000443 |
| DOID:3303 | notochordal cancer | 3/54 | 20/8007 | 0.000305 | 0.001044 | 0.000443 |
| DOID:2043 | hepatitis B | 7/54 | 197/8007 | 0.000326 | 0.001111 | 0.000472 |
| DOID:12930 | dilated cardiomyopathy | 5/54 | 93/8007 | 0.000383 | 0.001297 | 0.000551 |
| DOID:657 | adenoma | 9/54 | 340/8007 | 0.000395 | 0.001332 | 0.000565 |
| DOID:11123 | Henoch-Schoenlein purpura | 3/54 | 22/8007 | 0.000408 | 0.001338 | 0.000568 |
| DOID:13089 | intracranial arterial disease | 3/54 | 22/8007 | 0.000408 | 0.001338 | 0.000568 |
| DOID:1557 | hypersensitivity reaction type III disease | 3/54 | 22/8007 | 0.000408 | 0.001338 | 0.000568 |
| DOID:3527 | cerebral arterial disease | 3/54 | 22/8007 | 0.000408 | 0.001338 | 0.000568 |
| DOID:9809 | hypersensitivity vasculitis | 3/54 | 22/8007 | 0.000408 | 0.001338 | 0.000568 |
| DOID:4766 | embryoma | 9/54 | 352/8007 | 0.000509 | 0.001657 | 0.000703 |
| DOID:10652 | Alzheimer's disease | 10/54 | 430/8007 | 0.00051 | 0.001657 | 0.000703 |
| DOID:3526 | cerebral infarction | 4/54 | 56/8007 | 0.000523 | 0.00169 | 0.000717 |
| DOID:680 | tauopathy | 10/54 | 434/8007 | 0.000549 | 0.001764 | 0.000749 |
| DOID:3829 | pituitary adenoma | 4/54 | 58/8007 | 0.000599 | 0.001913 | 0.000812 |
| DOID:2893 | cervix carcinoma | 5/54 | 104/8007 | 0.000641 | 0.002037 | 0.000864 |
| DOID:4362 | cervical cancer | 5/54 | 105/8007 | 0.000669 | 0.002115 | 0.000898 |
| DOID:0050851 | glomerulosclerosis | 3/54 | 26/8007 | 0.000676 | 0.002115 | 0.000898 |
| DOID:1312 | focal segmental glomerulosclerosis | 3/54 | 26/8007 | 0.000676 | 0.002115 | 0.000898 |
| DOID:341 | peripheral vascular disease | 4/54 | 61/8007 | 0.000725 | 0.00226 | 0.000959 |
| DOID:13129 | severe pre-eclampsia | 3/54 | 27/8007 | 0.000756 | 0.002322 | 0.000985 |
| DOID:1724 | duodenal ulcer | 3/54 | 27/8007 | 0.000756 | 0.002322 | 0.000985 |
| DOID:2513 | basal cell carcinoma | 3/54 | 27/8007 | 0.000756 | 0.002322 | 0.000985 |
| DOID:13580 | cholestasis | 4/54 | 62/8007 | 0.000772 | 0.002357 | 0.001 |
| DOID:3371 | chondrosarcoma | 4/54 | 63/8007 | 0.00082 | 0.002492 | 0.001058 |
| DOID:1883 | hepatitis C | 7/54 | 233/8007 | 0.000891 | 0.002695 | 0.001144 |
| DOID:11963 | esophagitis | 3/54 | 29/8007 | 0.000936 | 0.002803 | 0.00119 |
| DOID:1712 | aortic valve stenosis | 3/54 | 29/8007 | 0.000936 | 0.002803 | 0.00119 |
| DOID:750 | peptic ulcer disease | 4/54 | 66/8007 | 0.000977 | 0.002913 | 0.001236 |
| DOID:4905 | pancreatic carcinoma | 7/54 | 237/8007 | 0.000985 | 0.002922 | 0.00124 |
| DOID:9970 | obesity | 8/54 | 313/8007 | 0.001079 | 0.003187 | 0.001353 |
| DOID:11394 | adult respiratory distress syndrome | 3/54 | 31/8007 | 0.00114 | 0.003351 | 0.001422 |
| DOID:2355 | anemia | 7/54 | 244/8007 | 0.001168 | 0.003415 | 0.001449 |
| DOID:784 | chronic kidney failure | 4/54 | 70/8007 | 0.001219 | 0.003535 | 0.0015 |
| DOID:1793 | pancreatic cancer | 8/54 | 319/8007 | 0.00122 | 0.003535 | 0.0015 |
| DOID:2797 | idiopathic interstitial pneumonia | 3/54 | 32/8007 | 0.001252 | 0.003612 | 0.001533 |
| DOID:654 | overnutrition | 8/54 | 322/8007 | 0.001296 | 0.003719 | 0.001579 |
| DOID:10825 | essential hypertension | 5/54 | 122/8007 | 0.001317 | 0.003764 | 0.001598 |
| DOID:0060121 | integumentary system benign neoplasm | 3/54 | 33/8007 | 0.001371 | 0.003882 | 0.001648 |
| DOID:3165 | skin benign neoplasm | 3/54 | 33/8007 | 0.001371 | 0.003882 | 0.001648 |
| DOID:0050736 | autosomal dominant disease | 9/54 | 409/8007 | 0.001486 | 0.004187 | 0.001777 |
| DOID:62 | aortic valve disease | 3/54 | 34/8007 | 0.001497 | 0.004199 | 0.001782 |
| DOID:1116 | pertussis | 3/54 | 35/8007 | 0.001629 | 0.00453 | 0.001923 |
| DOID:2596 | larynx cancer | 3/54 | 35/8007 | 0.001629 | 0.00453 | 0.001923 |
| DOID:3565 | meningioma | 4/54 | 77/8007 | 0.001737 | 0.004809 | 0.002041 |
| DOID:374 | nutrition disease | 8/54 | 338/8007 | 0.001765 | 0.00481 | 0.002042 |
| DOID:4928 | intrahepatic cholangiocarcinoma | 3/54 | 36/8007 | 0.001769 | 0.00481 | 0.002042 |
| DOID:5158 | pleural cancer | 3/54 | 36/8007 | 0.001769 | 0.00481 | 0.002042 |
| DOID:7474 | malignant pleural mesothelioma | 3/54 | 36/8007 | 0.001769 | 0.00481 | 0.002042 |
| DOID:255 | hemangioma | 3/54 | 37/8007 | 0.001916 | 0.005098 | 0.002164 |
| DOID:612 | primary immunodeficiency disease | 6/54 | 196/8007 | 0.001936 | 0.005098 | 0.002164 |
| DOID:13025 | retinopathy of prematurity | 2/54 | 10/8007 | 0.001941 | 0.005098 | 0.002164 |
| DOID:13949 | interstitial cystitis | 2/54 | 10/8007 | 0.001941 | 0.005098 | 0.002164 |
| DOID:1678 | chronic interstitial cystitis | 2/54 | 10/8007 | 0.001941 | 0.005098 | 0.002164 |
| DOID:2438 | dermis tumor | 2/54 | 10/8007 | 0.001941 | 0.005098 | 0.002164 |
| DOID:4418 | cutaneous fibrous histiocytoma | 2/54 | 10/8007 | 0.001941 | 0.005098 | 0.002164 |
| DOID:6536 | plasma cell neoplasm | 2/54 | 10/8007 | 0.001941 | 0.005098 | 0.002164 |
| DOID:3713 | ovary adenocarcinoma | 3/54 | 38/8007 | 0.00207 | 0.005393 | 0.002289 |
| DOID:9261 | nasopharynx carcinoma | 3/54 | 38/8007 | 0.00207 | 0.005393 | 0.002289 |
| DOID:4195 | hyperglycemia | 5/54 | 139/8007 | 0.002344 | 0.006076 | 0.002579 |
| DOID:13593 | eclampsia | 2/54 | 11/8007 | 0.002362 | 0.006076 | 0.002579 |
| DOID:3507 | dermatofibrosarcoma protuberans | 2/54 | 11/8007 | 0.002362 | 0.006076 | 0.002579 |
| DOID:0050339 | commensal bacterial infectious disease | 3/54 | 40/8007 | 0.002402 | 0.006153 | 0.002612 |
| DOID:4138 | bile duct disease | 4/54 | 86/8007 | 0.002607 | 0.006652 | 0.002823 |
| DOID:8857 | lupus erythematosus | 4/54 | 87/8007 | 0.002719 | 0.006881 | 0.002921 |
| DOID:9741 | biliary tract disease | 4/54 | 87/8007 | 0.002719 | 0.006881 | 0.002921 |
| DOID:11077 | brucellosis | 3/54 | 42/8007 | 0.002764 | 0.006967 | 0.002957 |
| DOID:5041 | esophageal cancer | 5/54 | 145/8007 | 0.002817 | 0.006998 | 0.00297 |
| DOID:11266 | hemorrhagic fever with renal syndrome | 2/54 | 12/8007 | 0.002822 | 0.006998 | 0.00297 |
| DOID:13359 | Ehlers-Danlos syndrome | 2/54 | 12/8007 | 0.002822 | 0.006998 | 0.00297 |
| DOID:1680 | chronic cystitis | 2/54 | 12/8007 | 0.002822 | 0.006998 | 0.00297 |
| DOID:1555 | urticaria | 3/54 | 44/8007 | 0.003159 | 0.007803 | 0.003312 |
| DOID:1307 | dementia | 5/54 | 150/8007 | 0.003262 | 0.008025 | 0.003406 |
| DOID:12895 | keratoconjunctivitis sicca | 2/54 | 13/8007 | 0.00332 | 0.008042 | 0.003413 |
| DOID:13608 | biliary atresia | 2/54 | 13/8007 | 0.00332 | 0.008042 | 0.003413 |
| DOID:288 | endometriosis of uterus | 2/54 | 13/8007 | 0.00332 | 0.008042 | 0.003413 |
| DOID:9368 | keratoconjunctivitis | 2/54 | 13/8007 | 0.00332 | 0.008042 | 0.003413 |
| DOID:3113 | papillary carcinoma | 3/54 | 45/8007 | 0.003369 | 0.008126 | 0.003449 |
| DOID:10155 | intestinal cancer | 7/54 | 300/8007 | 0.003773 | 0.009067 | 0.003849 |
| DOID:2913 | acute pancreatitis | 2/54 | 14/8007 | 0.003857 | 0.009168 | 0.003891 |
| DOID:1074 | kidney failure | 5/54 | 156/8007 | 0.003859 | 0.009168 | 0.003891 |
| DOID:399 | tuberculosis | 5/54 | 156/8007 | 0.003859 | 0.009168 | 0.003891 |
| DOID:381 | arthropathy | 4/54 | 97/8007 | 0.004028 | 0.009531 | 0.004046 |
| DOID:0060089 | endocrine organ benign neoplasm | 4/54 | 99/8007 | 0.004333 | 0.010214 | 0.004335 |
| DOID:2742 | auditory system disease | 4/54 | 100/8007 | 0.004491 | 0.010547 | 0.004477 |
| DOID:3376 | bone osteosarcoma | 3/54 | 50/8007 | 0.004544 | 0.010591 | 0.004495 |
| DOID:627 | severe combined immunodeficiency | 3/54 | 50/8007 | 0.004544 | 0.010591 | 0.004495 |
| DOID:4948 | gallbladder carcinoma | 3/54 | 51/8007 | 0.004805 | 0.011158 | 0.004736 |
| DOID:1679 | cystitis | 2/54 | 16/8007 | 0.005043 | 0.011526 | 0.004892 |
| DOID:1993 | rectum cancer | 2/54 | 16/8007 | 0.005043 | 0.011526 | 0.004892 |
| DOID:321 | tropical spastic paraparesis | 2/54 | 16/8007 | 0.005043 | 0.011526 | 0.004892 |
| DOID:3121 | gallbladder cancer | 3/54 | 52/8007 | 0.005075 | 0.011526 | 0.004892 |
| DOID:628 | combined T cell and B cell immunodeficiency | 3/54 | 52/8007 | 0.005075 | 0.011526 | 0.004892 |
| DOID:987 | alopecia | 3/54 | 52/8007 | 0.005075 | 0.011526 | 0.004892 |
| DOID:7166 | thyroiditis | 3/54 | 53/8007 | 0.005354 | 0.012115 | 0.005142 |
| DOID:2377 | multiple sclerosis | 5/54 | 169/8007 | 0.005419 | 0.012216 | 0.005185 |
| DOID:13378 | Kawasaki disease | 3/54 | 54/8007 | 0.005642 | 0.012552 | 0.005328 |
| DOID:2018 | hyperinsulinism | 3/54 | 54/8007 | 0.005642 | 0.012552 | 0.005328 |
| DOID:0050938 | breast lobular carcinoma | 2/54 | 17/8007 | 0.00569 | 0.012552 | 0.005328 |
| DOID:3457 | invasive lobular carcinoma | 2/54 | 17/8007 | 0.00569 | 0.012552 | 0.005328 |
| DOID:5395 | functioning pituitary adenoma | 2/54 | 17/8007 | 0.00569 | 0.012552 | 0.005328 |
| DOID:8923 | skin melanoma | 2/54 | 17/8007 | 0.00569 | 0.012552 | 0.005328 |
| DOID:9119 | acute myeloid leukemia | 4/54 | 107/8007 | 0.005709 | 0.012552 | 0.005328 |
| DOID:6050 | esophageal disease | 3/54 | 55/8007 | 0.00594 | 0.013013 | 0.005523 |
| DOID:3213 | demyelinating disease | 5/54 | 175/8007 | 0.006271 | 0.01369 | 0.005811 |
| DOID:365 | bladder disease | 2/54 | 18/8007 | 0.006374 | 0.013867 | 0.005886 |
| DOID:1602 | lymphadenitis | 3/54 | 58/8007 | 0.006887 | 0.014879 | 0.006315 |
| DOID:9942 | lymph node disease | 3/54 | 58/8007 | 0.006887 | 0.014879 | 0.006315 |
| DOID:1428 | endocrine pancreas disease | 3/54 | 59/8007 | 0.007222 | 0.015494 | 0.006576 |
| DOID:4535 | hypotrichosis | 3/54 | 59/8007 | 0.007222 | 0.015494 | 0.006576 |
| DOID:12252 | Cushing's syndrome | 2/54 | 20/8007 | 0.007848 | 0.01672 | 0.007097 |
| DOID:799 | varicose veins | 2/54 | 20/8007 | 0.007848 | 0.01672 | 0.007097 |
| DOID:8466 | retinal degeneration | 6/54 | 261/8007 | 0.007883 | 0.016737 | 0.007104 |
| DOID:11984 | hypertrophic cardiomyopathy | 3/54 | 62/8007 | 0.008284 | 0.017529 | 0.00744 |
| DOID:2723 | dermatitis | 5/54 | 189/8007 | 0.008621 | 0.018011 | 0.007645 |
| DOID:13133 | HELLP syndrome | 2/54 | 21/8007 | 0.008637 | 0.018011 | 0.007645 |
| DOID:3087 | gingivitis | 2/54 | 21/8007 | 0.008637 | 0.018011 | 0.007645 |
| DOID:3702 | cervical adenocarcinoma | 2/54 | 21/8007 | 0.008637 | 0.018011 | 0.007645 |
| DOID:715 | T-cell leukemia | 3/54 | 63/8007 | 0.008657 | 0.018011 | 0.007645 |
| DOID:421 | hair disease | 3/54 | 65/8007 | 0.009433 | 0.019484 | 0.00827 |
| DOID:10754 | otitis media | 2/54 | 22/8007 | 0.009459 | 0.019484 | 0.00827 |
| DOID:14221 | metabolic syndrome X | 2/54 | 22/8007 | 0.009459 | 0.019484 | 0.00827 |
| DOID:870 | neuropathy | 5/54 | 194/8007 | 0.009591 | 0.01969 | 0.008357 |
| DOID:11723 | Duchenne muscular dystrophy | 2/54 | 23/8007 | 0.010316 | 0.020901 | 0.008872 |
| DOID:4914 | esophagus adenocarcinoma | 2/54 | 23/8007 | 0.010316 | 0.020901 | 0.008872 |
| DOID:706 | mature B-cell neoplasm | 2/54 | 23/8007 | 0.010316 | 0.020901 | 0.008872 |
| DOID:707 | B-cell lymphoma | 2/54 | 23/8007 | 0.010316 | 0.020901 | 0.008872 |
| DOID:2452 | thrombophilia | 2/54 | 24/8007 | 0.011205 | 0.02263 | 0.009605 |
| DOID:219 | colon cancer | 6/54 | 282/8007 | 0.01131 | 0.022766 | 0.009663 |
| DOID:9256 | colorectal cancer | 6/54 | 287/8007 | 0.012261 | 0.024601 | 0.010442 |
| DOID:5672 | large intestine cancer | 6/54 | 288/8007 | 0.012458 | 0.024762 | 0.01051 |
| DOID:10871 | age related macular degeneration | 3/54 | 72/8007 | 0.012461 | 0.024762 | 0.01051 |
| DOID:2007 | degeneration of macula and posterior pole | 3/54 | 72/8007 | 0.012461 | 0.024762 | 0.01051 |
| DOID:3744 | cervical squamous cell carcinoma | 3/54 | 73/8007 | 0.012934 | 0.025425 | 0.010792 |
| DOID:6364 | migraine | 3/54 | 73/8007 | 0.012934 | 0.025425 | 0.010792 |
| DOID:0080015 | physical disorder | 4/54 | 136/8007 | 0.013071 | 0.025425 | 0.010792 |
| DOID:13809 | familial combined hyperlipidemia | 2/54 | 26/8007 | 0.013082 | 0.025425 | 0.010792 |
| DOID:205 | hyperostosis | 2/54 | 26/8007 | 0.013082 | 0.025425 | 0.010792 |
| DOID:2600 | laryngeal carcinoma | 2/54 | 26/8007 | 0.013082 | 0.025425 | 0.010792 |
| DOID:9471 | meningitis | 2/54 | 26/8007 | 0.013082 | 0.025425 | 0.010792 |
| DOID:4448 | macular degeneration | 3/54 | 74/8007 | 0.013417 | 0.025914 | 0.010999 |
| DOID:574 | peripheral nervous system disease | 3/54 | 74/8007 | 0.013417 | 0.025914 | 0.010999 |
| DOID:2978 | carbohydrate metabolic disorder | 2/54 | 27/8007 | 0.014068 | 0.027087 | 0.011497 |
| DOID:9778 | irritable bowel syndrome | 3/54 | 76/8007 | 0.014414 | 0.027668 | 0.011743 |
| DOID:1123 | spondyloarthropathy | 3/54 | 77/8007 | 0.014928 | 0.028478 | 0.012087 |
| DOID:76 | stomach disease | 3/54 | 77/8007 | 0.014928 | 0.028478 | 0.012087 |
| DOID:8692 | myeloid leukemia | 4/54 | 142/8007 | 0.015111 | 0.028739 | 0.012198 |
| DOID:0050735 | X-linked disease | 2/54 | 29/8007 | 0.016133 | 0.030496 | 0.012944 |
| DOID:4798 | aggressive systemic mastocytosis | 2/54 | 29/8007 | 0.016133 | 0.030496 | 0.012944 |
| DOID:9074 | systemic lupus erythematosus | 3/54 | 81/8007 | 0.017087 | 0.0322 | 0.013667 |
| DOID:3947 | adrenal gland hyperfunction | 2/54 | 30/8007 | 0.017212 | 0.032337 | 0.013725 |
| DOID:440 | neuromuscular disease | 3/54 | 83/8007 | 0.018229 | 0.034007 | 0.014434 |
| DOID:2583 | agammaglobulinemia | 2/54 | 31/8007 | 0.01832 | 0.034007 | 0.014434 |
| DOID:319 | spinal cord disease | 2/54 | 31/8007 | 0.01832 | 0.034007 | 0.014434 |
| DOID:620 | blood protein disease | 2/54 | 31/8007 | 0.01832 | 0.034007 | 0.014434 |
| DOID:13375 | temporal arteritis | 2/54 | 32/8007 | 0.019458 | 0.035904 | 0.01524 |
| DOID:525 | central nervous system vasculitis | 2/54 | 32/8007 | 0.019458 | 0.035904 | 0.01524 |
| DOID:0060115 | nervous system benign neoplasm | 2/54 | 33/8007 | 0.020625 | 0.03739 | 0.01587 |
| DOID:1483 | gingival disease | 2/54 | 33/8007 | 0.020625 | 0.03739 | 0.01587 |
| DOID:2115 | B cell deficiency | 2/54 | 33/8007 | 0.020625 | 0.03739 | 0.01587 |
| DOID:2228 | thrombocytosis | 2/54 | 33/8007 | 0.020625 | 0.03739 | 0.01587 |
| DOID:3277 | thymus cancer | 2/54 | 33/8007 | 0.020625 | 0.03739 | 0.01587 |
| DOID:3443 | mammary Paget's disease | 2/54 | 33/8007 | 0.020625 | 0.03739 | 0.01587 |
| DOID:0060058 | lymphoma | 3/54 | 88/8007 | 0.021266 | 0.03844 | 0.016316 |
| DOID:811 | lipodystrophy | 2/54 | 34/8007 | 0.02182 | 0.039327 | 0.016692 |
| DOID:50 | thyroid gland disease | 4/54 | 159/8007 | 0.02195 | 0.039447 | 0.016743 |
| DOID:10316 | pneumoconiosis | 2/54 | 35/8007 | 0.023044 | 0.041174 | 0.017476 |
| DOID:1272 | telangiectasis | 2/54 | 35/8007 | 0.023044 | 0.041174 | 0.017476 |
| DOID:3717 | gastric adenocarcinoma | 3/54 | 94/8007 | 0.025256 | 0.044996 | 0.019098 |
| DOID:349 | systemic mastocytosis | 2/54 | 38/8007 | 0.02688 | 0.047616 | 0.020211 |
| DOID:866 | vein disease | 2/54 | 38/8007 | 0.02688 | 0.047616 | 0.020211 |
| DOID:4866 | salivary gland adenoid cystic carcinoma | 2/54 | 40/8007 | 0.029571 | 0.052233 | 0.02217 |
| DOID:0060060 | non-Hodgkin lymphoma | 2/54 | 41/8007 | 0.030955 | 0.054523 | 0.023142 |
| DOID:9884 | muscular dystrophy | 3/54 | 103/8007 | 0.031947 | 0.056111 | 0.023816 |
| DOID:0050567 | orofacial cleft | 2/54 | 43/8007 | 0.033799 | 0.059029 | 0.025055 |
| DOID:674 | cleft palate | 2/54 | 43/8007 | 0.033799 | 0.059029 | 0.025055 |
| DOID:10603 | glucose intolerance | 2/54 | 44/8007 | 0.035258 | 0.061233 | 0.02599 |
| DOID:350 | mastocytosis | 2/54 | 44/8007 | 0.035258 | 0.061233 | 0.02599 |
| DOID:11830 | myopia | 2/54 | 45/8007 | 0.036741 | 0.063453 | 0.026933 |
| DOID:3458 | breast adenocarcinoma | 2/54 | 45/8007 | 0.036741 | 0.063453 | 0.026933 |
| DOID:1686 | glaucoma | 3/54 | 109/8007 | 0.036876 | 0.063508 | 0.026956 |
| DOID:3324 | mood disorder | 4/54 | 189/8007 | 0.038066 | 0.065148 | 0.027652 |
| DOID:161 | keratosis | 2/54 | 46/8007 | 0.038248 | 0.065148 | 0.027652 |
| DOID:3480 | uveal disease | 2/54 | 46/8007 | 0.038248 | 0.065148 | 0.027652 |
| DOID:3952 | adrenal cortex disease | 2/54 | 46/8007 | 0.038248 | 0.065148 | 0.027652 |
| DOID:9553 | adrenal gland disease | 2/54 | 47/8007 | 0.039779 | 0.06757 | 0.02868 |
| DOID:0050624 | gastrointestinal system benign neoplasm | 2/54 | 50/8007 | 0.044507 | 0.075395 | 0.032001 |
| DOID:1826 | epilepsy syndrome | 4/54 | 201/8007 | 0.046013 | 0.077733 | 0.032994 |
| DOID:1205 | hypersensitivity reaction type I disease | 3/54 | 122/8007 | 0.048813 | 0.08224 | 0.034907 |
| DOID:5223 | infertility | 4/54 | 206/8007 | 0.04958 | 0.083305 | 0.035359 |
| DOID:1107 | esophageal carcinoma | 3/54 | 123/8007 | 0.049802 | 0.083451 | 0.035421 |
