# Supplementary material for: Application of an angiogenesis-related genes risk model in lung adenocarcinoma prognosis and immunotherapy
Source: Front Genet. 2023 Feb 1;14:1092968. doi: 10.3389/fgene.2023.1092968 (PMC9929558; doi:10.3389/fgene.2023.1092968)
Supplement: Supplementary file 7 [file Table2.DOCX]

Table S2 The information of drugs

| Gene | Drug | cor | pvalue |
| --- | --- | --- | --- |
| PECAM1 | Zalcitabine | 0.904364 | 4.09E-23 |
| PECAM1 | Nelarabine | 0.867834 | 2.88E-19 |
| PECAM1 | Methylprednisolone | 0.649552 | 1.97E-08 |
| PECAM1 | Hydroxychloroquine Sulfate | 0.626906 | 8.37E-08 |
| PECAM1 | Bendamustine | 0.602289 | 3.56E-07 |
| MET | auranofin | -0.56371 | 2.73E-06 |
| CCND2 | Amiodarone hydrochloride | 0.555821 | 4.02E-06 |
| PECAM1 | Ergosterol | 0.543062 | 7.36E-06 |
| PECAM1 | Asparaginase | 0.539035 | 8.85E-06 |
| MET | Lomustine | -0.53265 | 1.18E-05 |
| POSTN | Zoledronate | 0.529286 | 1.37E-05 |
| PECAM1 | Chlorambucil | 0.493745 | 6.10E-05 |
| MET | Arsenic trioxide | -0.48507 | 8.57E-05 |
| POSTN | Caffeic acid | 0.478533 | 0.00011 |
| PECAM1 | Ribavirin | 0.477004 | 0.000117 |
| PECAM1 | Fluphenazine | 0.473941 | 0.000131 |
| PECAM1 | Hydroxyurea | 0.468512 | 0.00016 |
| CCND2 | Artemether | 0.4685 | 0.00016 |
| MET | Carmustine | -0.46412 | 0.000188 |
| PECAM1 | Arsenic trioxide | 0.459861 | 0.000219 |
| PECAM1 | Ifosfamide | 0.454342 | 0.000266 |
| MET | BLU-667 | 0.452475 | 0.000284 |
| PDGFB | Selumetinib | -0.45026 | 0.000307 |
| PECAM1 | Pipobroman | 0.449215 | 0.000318 |
| PDGFB | Pipamperone | -0.44792 | 0.000332 |
| PECAM1 | Uracil mustard | 0.447813 | 0.000334 |
| PECAM1 | Melphalan | 0.445659 | 0.000359 |
| CCND2 | uridin | 0.443487 | 0.000387 |
| MET | Artemether | -0.4423 | 0.000403 |
| CCND2 | Nelfinavir | 0.436682 | 0.000486 |
| TIMP1 | Simvastatin | 0.436518 | 0.000489 |
| CCND2 | Econazole nitrate | 0.435596 | 0.000504 |
| PDGFB | Erlotinib | 0.432032 | 0.000567 |
| PECAM1 | Fludarabine | 0.427074 | 0.000666 |
| PDGFB | Afatinib | 0.423823 | 0.00074 |
| MET | Pipobroman | -0.41836 | 0.00088 |
| PDGFB | Ibrutinib | 0.415917 | 0.00095 |
| PDGFB | Vinblastine | -0.41528 | 0.000969 |
| MET | IDOXURIDINE | -0.4143 | 0.000999 |
| PDGFB | Dacomitinib | 0.4129 | 0.001043 |
| PECAM1 | Masoprocol | 0.409086 | 0.001173 |
| MET | ciclosporin | -0.40716 | 0.001244 |
| PECAM1 | Dexamethasone Decadron | 0.405593 | 0.001304 |
| PECAM1 | Thiotepa | 0.404369 | 0.001354 |
| PECAM1 | Triethylenemelamine | 0.403755 | 0.001379 |
| CCND2 | Amuno | 0.402298 | 0.00144 |
| PDGFB | zanubrutinib | 0.402149 | 0.001447 |
| PECAM1 | IDOXURIDINE | 0.401339 | 0.001482 |
| PDGFB | Everolimus | 0.399925 | 0.001546 |
| CCND2 | ciclosporin | 0.399785 | 0.001553 |
| MET | Ifosfamide | -0.39843 | 0.001616 |
| CCND2 | Alectinib | 0.397933 | 0.00164 |
| PDGFB | aloin | 0.396928 | 0.00169 |
| PECAM1 | Etoposide | 0.396858 | 0.001693 |
| PECAM1 | DACARBAZINE | 0.393609 | 0.001862 |
| MET | Etoposide | -0.39302 | 0.001894 |
| PECAM1 | Artemether | 0.389511 | 0.002097 |
| CCND2 | Artesunate | 0.389212 | 0.002115 |
| CCND2 | rifa | 0.388253 | 0.002174 |
| TIMP1 | Lovastatin | 0.388014 | 0.002189 |
| PDGFB | TYROTHRICIN | -0.38749 | 0.002222 |
| PDGFB | Osimertinib | 0.387022 | 0.002252 |
| CCND2 | Irofulven | -0.38609 | 0.002313 |
| MET | Irofulven | 0.385043 | 0.002383 |
| PDGFB | PENTOSTATIN | 0.384276 | 0.002435 |
| MET | Chlorambucil | -0.38396 | 0.002457 |
| PECAM1 | Carmustine | 0.382324 | 0.002573 |
| POSTN | BLU-667 | 0.382198 | 0.002582 |
| MET | Zalcitabine | -0.38144 | 0.002638 |
| CCND2 | Imatinib | 0.379693 | 0.00277 |
| MET | Fulvestrant | -0.37802 | 0.002903 |
| PECAM1 | Idarubicin | 0.375659 | 0.003099 |
| MET | Isotretinoin | -0.37564 | 0.0031 |
| PDGFB | Crizotinib | -0.37551 | 0.003111 |
| PDGFB | Paclitaxel | -0.37505 | 0.003151 |
| CCND2 | Isotretinoin | 0.374283 | 0.003218 |
| PECAM1 | pentamidine isethionate | -0.37135 | 0.003487 |
| PDGFB | Neratinib | 0.371047 | 0.003516 |
| TIMP1 | Hydroxychloroquine Sulfate | -0.37066 | 0.003553 |
| MET | Carboplatin | -0.36821 | 0.003796 |
| PDGFB | ARRY-162 | -0.36747 | 0.003873 |
| PECAM1 | Cytarabine | 0.36699 | 0.003923 |
| MET | Masoprocol | -0.36663 | 0.003961 |
| MET | Tamoxifen | -0.3664 | 0.003986 |
| CCND2 | Denileukin Diftitox Ontak | 0.365085 | 0.004129 |
| PECAM1 | Calusterone | 0.364827 | 0.004157 |
| MET | Cyclophosphamide | -0.36267 | 0.004402 |
| CCND2 | Celecoxib | 0.362596 | 0.004411 |
| MET | Noscapine | -0.36222 | 0.004455 |
| PECAM1 | Cortivazol | 0.36018 | 0.004702 |
| CCND2 | NMS-E628 | 0.357225 | 0.00508 |
| MET | Melphalan | -0.35685 | 0.00513 |
| MET | Uracil mustard | -0.35493 | 0.005392 |
| MET | Hydroxychloroquine Sulfate | -0.35469 | 0.005425 |
| MET | VINORELBINE | -0.35445 | 0.005459 |
| PECAM1 | LEE-011 | 0.353872 | 0.005541 |
| PECAM1 | Oxaliplatin | 0.353496 | 0.005595 |
| PDGFB | ABT-199 | -0.35255 | 0.005733 |
| CCND2 | LDK-378 | 0.35237 | 0.005759 |
| PDGFB | PENTAMIDINE ISETHIONATE | -0.35199 | 0.005816 |
| PDGFB | Vincristine | -0.35126 | 0.005925 |
| PECAM1 | Valrubicin | 0.351199 | 0.005934 |
| CCND2 | Dromostanolone Propionate | 0.349517 | 0.006194 |
| MET | Curcumin | -0.34895 | 0.006284 |
| PDGFB | Vemurafenib | -0.34859 | 0.006341 |
| PDGFB | Carfilzomib | -0.34757 | 0.006508 |
| PECAM1 | 6-THIOGUANINE | 0.347411 | 0.006534 |
| PECAM1 | Cyclophosphamide | 0.343945 | 0.007129 |
| CCND2 | Cyclophosphamide | 0.343693 | 0.007174 |
| TIMP1 | Fulvestrant | -0.34323 | 0.007256 |
| MET | DACARBAZINE | -0.34306 | 0.007288 |
| PDGFB | Depsipeptide | -0.34257 | 0.007377 |
| PECAM1 | Palbociclib | 0.340954 | 0.007679 |
| CCND2 | Pimozide | 0.340584 | 0.00775 |
| MET | LY-2835219 | 0.340437 | 0.007778 |
| PECAM1 | Teniposide | 0.339826 | 0.007896 |
| PECAM1 | Dexrazoxane | 0.33889 | 0.008081 |
| PDGFB | Cobimetinib (isomer 1) | -0.33794 | 0.008271 |
| PECAM1 | Lomustine | 0.336936 | 0.008477 |
| PECAM1 | Irofulven | -0.33649 | 0.00857 |
| MET | Dasatinib | 0.336245 | 0.008621 |
| CCND2 | Crizotinib | 0.335877 | 0.008699 |
| CCND2 | claritin | 0.335738 | 0.008729 |
| TIMP1 | Oxaliplatin | -0.33413 | 0.009076 |
| TIMP1 | Bendamustine | -0.33263 | 0.009411 |
| PDGFB | Eribulin mesilate | -0.33155 | 0.009658 |
| PECAM1 | ANCITABINE HYDROCHLORIDE | 0.331071 | 0.009771 |
| MET | Paclitaxel | -0.33037 | 0.009936 |
| MET | 6-MERCAPTOPURINE | -0.32679 | 0.010822 |
| TIMP1 | Cabozantinib | 0.324569 | 0.011402 |
| MET | Simvastatin | 0.324137 | 0.011519 |
| MET | Pipamperone | -0.32348 | 0.011697 |
| POSTN | Allopurinol | -0.32348 | 0.011698 |
| PECAM1 | Vorinostat | 0.321958 | 0.012121 |
| PDGFB | Vinorelbine | -0.32073 | 0.012473 |
| PDGFB | Lapatinib | 0.320584 | 0.012515 |
| MET | Clotrimazole | -0.31971 | 0.012769 |
| POSTN | Noscapine | 0.319696 | 0.012775 |
| MET | Estramustine | -0.31963 | 0.012795 |
| MET | Teniposide | -0.31915 | 0.012936 |
| POSTN | Rapamycin | 0.318712 | 0.013068 |
| MET | Ixabepilone | -0.3178 | 0.013345 |
| MET | Fluphenazine | -0.31674 | 0.013675 |
| PECAM1 | Mitoxantrone | 0.315376 | 0.014108 |
| PECAM1 | Noscapine | 0.313908 | 0.014588 |
| PDGFB | COLCHICINE | -0.31238 | 0.015101 |
| PECAM1 | Cladribine | 0.31225 | 0.015146 |
| TIMP1 | Palbociclib | -0.31208 | 0.015203 |
| MET | Mebendazole | -0.3106 | 0.01572 |
| POSTN | Nitrogen mustard | -0.31057 | 0.01573 |
| MET | Triethylenemelamine | -0.31049 | 0.015757 |
| MET | Ergosterol | -0.30933 | 0.016173 |
| PDGFB | Actinomycin D | -0.30883 | 0.016358 |
| MET | Raloxifene | -0.3088 | 0.016368 |
| MET | Nilotinib | -0.30815 | 0.016608 |
| CCND2 | Hydroxyurea | 0.308055 | 0.016641 |
| PECAM1 | Irinotecan | 0.306797 | 0.017114 |
| MET | Imatinib | -0.30619 | 0.017345 |
| MET | Lovastatin | 0.305214 | 0.017724 |
| PECAM1 | DIGOXIN | 0.304115 | 0.018159 |
| POSTN | RAPAMYCIN | 0.303522 | 0.018397 |
| PDGFB | Vandetanib | 0.302556 | 0.018791 |
| MET | Thiotepa | -0.30237 | 0.018867 |
| PECAM1 | Daunorubicin | 0.301757 | 0.019122 |
| POSTN | Temsirolimus | 0.299649 | 0.02002 |
| MET | ETHINYL ESTRADIOL | -0.29876 | 0.02041 |
| MET | Nelarabine | -0.29842 | 0.020561 |
| MET | rifa | -0.29813 | 0.020687 |
| PDGFB | Colchicine | -0.2975 | 0.020973 |
| PECAM1 | Epirubicin | 0.297425 | 0.021006 |
| MET | Hydroxyurea | -0.29725 | 0.021087 |
| PECAM1 | M-AMSA | 0.296068 | 0.021627 |
| TIMP1 | Irofulven | 0.295885 | 0.021712 |
| PDGFB | Gefitinib | 0.2952 | 0.022033 |
| PECAM1 | Nitrogen mustard | 0.29489 | 0.022179 |
| CCND2 | DACARBAZINE | 0.294788 | 0.022228 |
| PDGFB | Mitotane | 0.293879 | 0.022663 |
| PDGFB | Bortezomib | -0.29284 | 0.023167 |
| PDGFB | Homoharringtonine | -0.29165 | 0.02376 |
| PECAM1 | Haloperidol | -0.29151 | 0.02383 |
| MET | Vorinostat | -0.29115 | 0.024013 |
| CCND2 | leflunomide | 0.290361 | 0.024415 |
| MET | Oxaliplatin | -0.2901 | 0.024547 |
| PECAM1 | DAUNORUBICIN | 0.289562 | 0.024828 |
| MET | 6-THIOGUANINE | -0.2893 | 0.024963 |
| MET | Dexrazoxane | -0.28907 | 0.025085 |
| MET | Sonidegib | 0.288361 | 0.02546 |
| POSTN | Cabozantinib | 0.287026 | 0.02618 |
| MET | Trametinib | 0.28698 | 0.026204 |
| MET | Fluvastatin | 0.28615 | 0.026661 |
| PECAM1 | ciclosporin | 0.285387 | 0.027085 |
| MET | ANCITABINE HYDROCHLORIDE | -0.28308 | 0.028405 |
| PECAM1 | auranofin | 0.282676 | 0.028642 |
| CCND2 | ARTENIMOL | 0.281574 | 0.029295 |
| TIMP1 | Fluvastatin | 0.281291 | 0.029465 |
| PDGFB | Cabozantinib | -0.28102 | 0.02963 |
| CCND2 | Nilotinib | 0.280892 | 0.029706 |
| MET | Parthenolide | -0.28081 | 0.029756 |
| PDGFB | umbralisib | 0.280506 | 0.029941 |
| PDGFB | Trametinib | -0.28043 | 0.029989 |
| CCND2 | BLU-285 | 0.279561 | 0.030522 |
| PDGFB | STREPTOZOCIN | 0.279278 | 0.030698 |
| CCND2 | Carmustine | 0.279103 | 0.030807 |
| TIMP1 | Isotretinoin | -0.2788 | 0.030996 |
| PECAM1 | Ancitabine hydrochloride | 0.278458 | 0.031212 |
| CCND2 | Fluphenazine | 0.278261 | 0.031337 |
| TIMP1 | Midostaurin | 0.277648 | 0.031727 |
| POSTN | Intedanib | 0.277309 | 0.031945 |
| MET | Midostaurin | 0.276848 | 0.032243 |
| CCND2 | Sulfatinib | 0.275574 | 0.033078 |
| CCND2 | timazid | 0.27507 | 0.033413 |
| MET | Raltitrexed | -0.27492 | 0.033515 |
| PDGFB | Dabrafenib | -0.27399 | 0.03414 |
| MET | Bendamustine | -0.27353 | 0.034459 |
| CCND2 | PF-06463922 | 0.273346 | 0.034582 |
| MET | Denileukin Diftitox Ontak | -0.27283 | 0.03494 |
| CCND2 | brigatinib | 0.272103 | 0.035446 |
| PECAM1 | Raltitrexed | 0.270255 | 0.036763 |
| MET | Nelfinavir | -0.26954 | 0.037284 |
| POSTN | Osimertinib | -0.26903 | 0.03766 |
| PECAM1 | OUABAIN | 0.268564 | 0.038004 |
| CCND2 | artesunate | 0.266961 | 0.039212 |
| MET | melbex | -0.26547 | 0.04036 |
| POSTN | Midostaurin | 0.265381 | 0.040433 |
| MET | timazid | -0.26507 | 0.040676 |
| PDGFB | olmutinib | 0.264519 | 0.041113 |
| TIMP1 | Raloxifene | -0.26394 | 0.041576 |
| CCND2 | Megestrol acetate | 0.263238 | 0.04214 |
| PDGFB | auranofin | -0.26311 | 0.042242 |
| TIMP1 | Fluorouracil | -0.26272 | 0.042561 |
| PECAM1 | Carboplatin | 0.262552 | 0.042699 |
| PECAM1 | Acrichine | 0.262513 | 0.042731 |
| PDGFB | Carmustine | -0.26239 | 0.042836 |
| MET | Nitrogen mustard | -0.26196 | 0.043185 |
| MET | ARSENIC TRIOXIDE | -0.26135 | 0.043694 |
| POSTN | Parthenolide | -0.26087 | 0.044092 |
| MET | Epirubicin | -0.26085 | 0.044108 |
| TIMP1 | Zalcitabine | -0.25892 | 0.045764 |
| PDGFB | Noscapine | -0.2588 | 0.045861 |
| PDGFB | pentamidine isethionate | -0.25849 | 0.046137 |
| PECAM1 | Voreloxin | 0.258273 | 0.046324 |
| POSTN | Dasatinib | 0.258253 | 0.046341 |
| MET | Axitinib | -0.25737 | 0.047116 |
| PECAM1 | Artesunate | 0.257372 | 0.047118 |
| MET | DECITABINE | -0.257 | 0.047451 |
| PDGFB | Encorafenib | -0.25676 | 0.047667 |
| TIMP1 | Ifosfamide | -0.25514 | 0.049132 |
| PDGFB | ARRY-380 | 0.254635 | 0.0496 |
| PDGFB | ETHINYL ESTRADIOL | -0.25463 | 0.049603 |
