# Supplementary material for: Application of an angiogenesis-related genes risk model in lung adenocarcinoma prognosis and immunotherapy
Source: Front Genet. 2023 Feb 1;14:1092968. doi: 10.3389/fgene.2023.1092968 (PMC9929558; doi:10.3389/fgene.2023.1092968)
Supplement: Supplementary file 8 [file Table3.DOCX]

| Table S3 The primer information for model genes | | |
| --- | --- | --- |
| Genes | Forward Primer sequence | Reverse Primer sequence |
| MET | AGCAATGGGGAGTGTAAAGAGG | CCCAGTCTTGTACTCAGCAAC |
| CCND2 | TGTGCATTTACACCGACAAC | ATCATCGACGGTGGGTACAT |
| TIMP1 | GCTTCTGGCATCCTGTTGTT | TTTGCAGGGGATGGATAAAC |
| POSTN | GCGAGATCATCAAGCCAGCAGAG | ATGTCCAGTCTCCAGGTTGTGTCA |
| PDGFB | TGCTGAGTGACCACTCGATC | TCCAAGGGTCTCCTTCAGTG |
| PECAM1 | TTCACCAAGATAGCCTCAAAGTCG | TGGGAGAGCATTTCACATACGACT |
