# Supplementary material for: Application of an angiogenesis-related genes risk model in lung adenocarcinoma prognosis and immunotherapy
Source: Front Genet. 2023 Feb 1;14:1092968. doi: 10.3389/fgene.2023.1092968 (PMC9929558; doi:10.3389/fgene.2023.1092968)
Supplement: Supplementary file 9 [file Table4.DOCX]

| Table S4 The information of model comparison | | |
| --- | --- | --- |
| Literature | Characteristics | C - index |
| Present study | Angiogenesis | 0.644 |
| Jin_Duan_2021 | Pyroptosis | 0.627 |
| Wei_Zhang_2022 | Pyroptosis | 0.625 |
| Jian_Yang_2022 | Cell cycle checkpoints | 0.61 |
| Qian_Xu_2021 | Aging | 0.61 |
| Zetian_Gong_2022 | Methylation | 0.588 |
| Wen_Yu_Zhai_2022 | Inflammatory | 0.587 |
